# Mechanistic Insights into Lewis Acid-Catalyzed Formal [3 + 2] Cycloadditions of Aziridines: A Molecular Electron Density Theory Study

**DOI:** 10.3390/molecules31030509

**Published:** 2026-02-02

**Authors:** Luis R. Domingo, Patricia Pérez, Maria José Aurell

**Affiliations:** 1 Independent Researcher, Av. Tirso de Molina 20, 46015 Valencia, Spain; 2 Facultad de Ciencias, Escuela de Química y Farmacia, Universidad San Sebastián, Campus Ciudad Universitaria, Av. del Cóndor 720, Ciudad Empresarial, Huechuraba, Santiago 8580704, Chile; 3 Department of Organic Chemistry, University of Valencia, Dr. Moliner 50, 46100 Burjassot, Spain; maria.j.aurell@uv.es

**Keywords:** aziridines, oxazolidines, Lewis acids, formal [3 + 2] cycloadditions, nucleophilic substitution reactions, Molecular Electron Density Theory, relative interacting atomic energy analysis

## Abstract

The Lewis acid (LA)-promoted formal [3 + 2] cycloaddition (32CA) reaction of 2-phenyl-1-tosylaziridine (2PTA) with ketone has been studied within the framework of Molecular Electron Density Theory (MEDT) at the *ω*B97X-D/6-311G(d,p) computational level in dichloromethane. This formal 32CA reaction proceeds through a stepwise mechanism, involving an initial BF_3_ LA-promoted aziridine ring-opening process, followed by a ring-closure process to yield the 1,3-oxazolidine product. The activation enthalpy of the most favorable C2–N1 breaking bond step, ΔH^≠^ = 6.42 kcal·mol^−1^, is 20.98 kcal·mol^−1^ lower than that of the non-catalyzed process, the aziridine ring-opening process being totally C2 regioselective and stereospecific. A topological analysis of the electron localization function (ELF) reveals that the most favorable transition state structure exhibits C2 carbocationic character; in this structure, the C2–N1 single bond has broken, while the C2–O4 single bond has not yet formed. A relative interacting atomic energy (RIAE) analysis of the aziridine ring-opening step reveals that the stabilization of the sulfonamide/LA leaving group and that of the ketone frameworks are the key factors responsible for the reduction in the activation barrier in the presence of LAs. LAs shift the mechanism of the aziridine ring-opening process from S_N_2-like in the non-catalyzed reaction to S_N_1-like in the LA-promoted process, which occurs with the inversion of the C2 carbon.

## 1. Introduction

**Chart 1:**
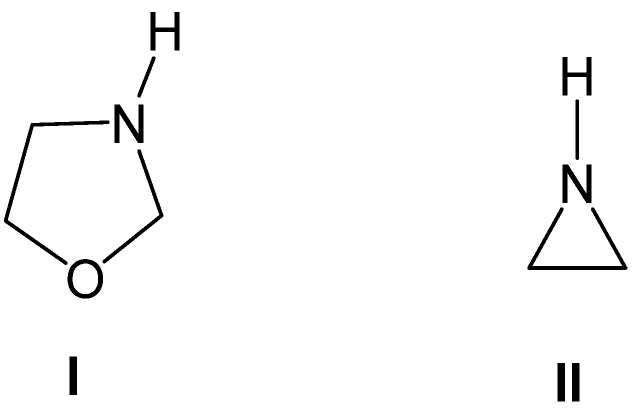
Structures of the simplest 1,3-oxazolidine **I** and aziridine **II**.

1,3-Oxazolidines (see the structure of the simplest 1,3-oxazolidine **I** in Chart 1) are five-membered heterocycles that exhibit diverse biological activities, including antibacterial, antimicrobial, and antitumor properties [1,2]. These heterocyclic compounds are also valuable as synthetic intermediates [3,4,5,6], chiral auxiliaries [7,8,9], and precursors of synthetically and pharmaceutically important 1,2-amino alcohols [10,11,12,13], which are structural motifs found in numerous natural products [14,15,16].

**Scheme 1:**
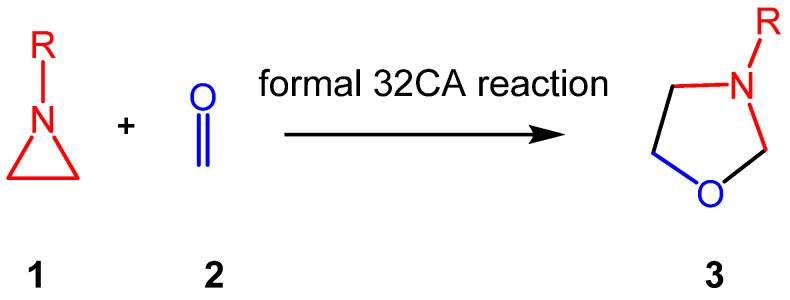
Synthesis of 1,3-oxazolidines **3** via formal 32CA reactions of aziridines **1** with carbonyl compounds **2**.

Aziridines constitute an important class of aza-three-membered heterocyclic compounds in organic synthesis, valued for their biological significance and remarkable synthetic versatility as multifunctional building blocks [17]. The structure of the simplest aziridine **II** is shown in Chart 1. Aziridines are commonly employed in the synthesis of oxazolidines **I**, acting as a three-atom-component in formal [3 + 2] cycloaddition (32CA) reactions with carbonyl compounds (see Scheme 1).

**Scheme 2:**
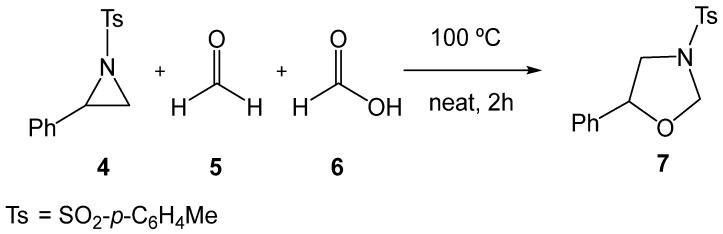
Catalyst-free formal 32CA reaction of 2PTA **4** with formaldehyde **5** in the presence of formic acid **6**, yielding the oxazolidine **7**.

Several experimental studies have been reported on the synthesis of oxazolidines **3** through formal 32CA reactions of aziridines **1**. In 2018, Majee et al. [18] reported the catalyst-free tandem ring-opening/ring-closing reaction of 2-phenyl-1-tosylaziridine (2PTA, **4**) with formaldehyde **5** in the presence of formic acid **6**, yielding the oxazolidine **7** (see Scheme 2). This catalyst-free reaction required heating at 100 °C to promote the opening of the aziridine ring.

**Scheme 3:**
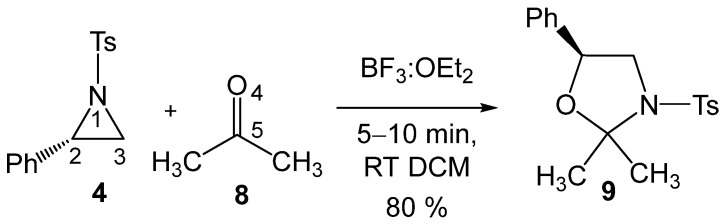
BF_3_ LA-catalyzed formal 32CA reaction of 2PTA **4** with ketone **8**, yielding 1,3-oxazolidine **9**.

These formal 32CA reactions require the presence of Lewis acids (LAs) as a catalyst for the aziridine ring-opening step, allowing these tandem reactions to occur at room temperature (RT) and in short reaction times. Thus, Ghorai and Ghosh [19] explored the S_N_2-type ring-opening of 2PTA **4** with ketone **8** in the presence of BF_3_ as an LA catalyst, yielding, within a few minutes at RT in dichloromethane (DCM), the corresponding 1,3-oxazolidine **9** in 80% yield (see Scheme 3). This formal 32CA reaction was completely C2 regioselective and stereospecific.

**Scheme 4:**
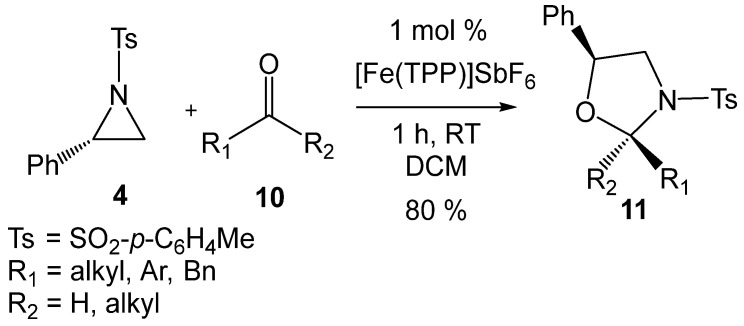
[Fe(TPP)]SbF_6_ LA-catalyzed formal 32CA reaction of 2PTA **4** with aldehydes and ketones **10** yielding 1,3-oxazolidines **11**.

In 2019, Matsubara et al. [20] reported an efficient iron-porphyrin LA-catalyzed formal 32CA reaction of 2PTA **4** with carbonyl compounds **10** affording 1,3-oxazolidines **11** with high regio- and diastereoselectivity (see Scheme 4). The requirement for LA catalysis in these tandem ring-opening/ring-closing reactions to occur under mild conditions strongly suggests that the first aziridine ring-opening process is the rate-determining step of these tandem processes. Moreover, the formation of non-racemic products in these LA-catalyzed formal 32CA reactions (see Scheme 3 and Scheme 4) strongly provides compelling evidence that the aziridine ring-opening processes proceed via a substitution reaction (SN) consistent with an S_N_2-type mechanism [19,21].

**Scheme 5:**
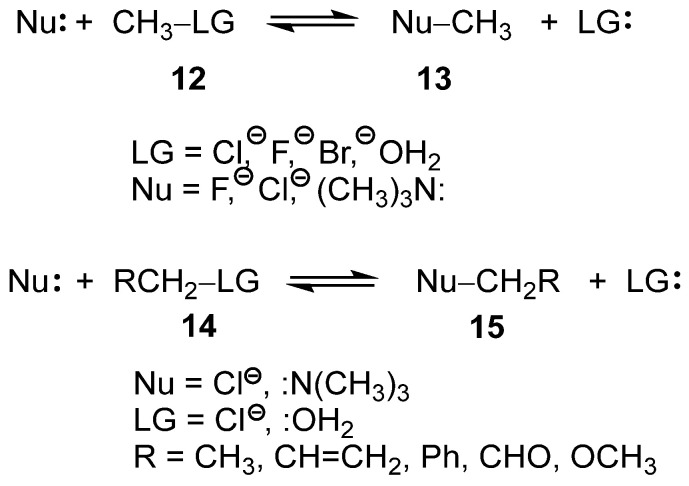
SN reactions of mono and di-substituted methyl derivatives **12** and **14**.

Recently, the SN reactions of the mono- and di-substituted methyl derivatives **12** and **14**, respectively, have been studied within the framework of Molecular Electron Density Theory [22] (MEDT) (see Scheme 5) [23,24]. These studies permitted to obtain several insightful conclusions: (i) neither the classical unimolecular (S_N_1) nor bimolecular (S_N_2) SN mechanisms [21] were identified for any of the reactions studied; (ii) in all analyzed SN reactions, the carbon–leaving group (LG) single bonds were already broken at the transition state structures (TSs), while the formation of the new carbon–nucleophile (Nu) single bonds had not yet begun; (iii) consequently, the central carbon at the TS adopts a trigonal-planar geometry with pronounced carbocation character [23]; (iv) such behavior requires the close proximity of both Nu and LG groups to stabilize the carbocation center through a global electron density transfer [25] (GEDT); and finally, (v) the presence of excellent LGs such as –OH_2_, characterized by a high nucleofugality Λ index [24,26], Λ = 1.34 eV, reduces the participation of the Nu in a S_N_1-like mechanism, while the use of poor LGs, such as –Cl, Λ = 0.77 eV [24], demands the participation of the Nu to stabilize the central carbocation in an S_N_2-like mechanism [24].

**Scheme 6:**
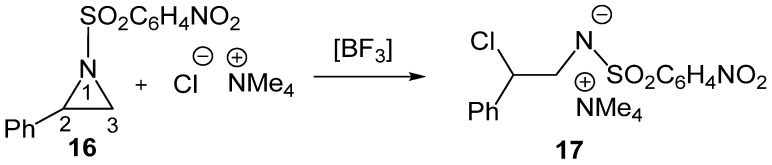
BF_3_ LA-catalyzed C2 regioselective aziridine ring-opening process of 2PNSA **16** by chloride anion Cl^−^ in the presence of the counterion NMe_4_^+^.

To gain deeper insight into the electronic effects of LAs on SN reactions, the LA-catalyzed regioselective aziridine ring-opening of 2-phenyl-1-*p*-nitrobenzenesulfonylaziridine (2PNSA, **16**) by the chloride anion Cl^−^ as Nu, in the presence of the counterion NMe_4_^+^, experimentally reported by Ghorai et al. [27], was very recently studied within the framework of the MEDT [28] (see Scheme 6).

That MEDT study yielded several relevant conclusions regarding the LA-catalyzed aziridine ring-opening processes: (i) the BF_3_ LA reduces the activation enthalpy of the non-catalyzed process by more than 17 kcal·mol^−1^, rendering the ring-opening of 2-phenylaziridine **16** completely C2 regioselective; (ii) topological analyses of the electron density at the TSs revealed that, while the aziridine C2–N1 single bond involving the sulfonamides LG had already cleaved, the new C2–Cl single bond involving the chloride anion Cl^−^ Nu had not yet begun, with the central C2 carbon exhibiting a trigonal planar carbocation structure; (iii) a relative interacting atomic energy [29] (RIAE) analysis of the LA-catalyzed aziridine ring-opening of a simplest 1-methanesulfonylaziridine **18** by chloride anion Cl^−^ as Nu in the presence of the counterion NMe_4_^+^ demonstrated that stabilization of both the sulfonamide: LA LG and the nucleophilic chloride anion Cl^−^ are the dominant factors responsible for lowering the activation barrier, which decrease with the acidic character of the LA, BH_3_ < BF_3_ < AlCl_3_; and finally, (iv) the increase in the acidic character of the LA shifts the aziridine ring-opening process from an S_N_2-like to a more S_N_1-like mechanism, thereby reducing the Nu participation.

The recent MEDT studies of SN reactions involving substituted primary carbons have led to two major conclusions [23,24]: (i) although these organic reactions involve the replacement of an atom or group of atoms, named the LG, by another atom or group of atoms, named the nucleophile, Nu, topological and energetic analyses of S_N_2-type reactions suggest that the activation barrier is primarily governed by the departure of the LG, whereas the contribution of the Nu is secondary in a S_N_2-like mechanism; and (ii) the involvement of highly efficient LGs reduces the participation of the Nu, thereby shifting the mechanism to an S_N_1-like mechanism.

The BF_3_ LA-promoted formal 32CA reaction of 2PTA **4** with ketone **8**, yielding 1,3-oxazolidine **9**, experimentally studied by Ghorai and Ghosh [19] (see Scheme 3), is herein studied within the framework of the MEDT at the *ω*B97X-D/6-311G(d,p) computational level. This study aims to provide meaningful insights into the participation of aziridines in the synthesis of 1,3-oxazolidines (see Scheme 1, Scheme 2, Scheme 3 and Scheme 4). The specific role of the BF_3_ LA in promoting the aziridine ring-opening process in the presence of a ketone, which enables these formal 32CA reactions, is analyzed in detail. Finally, a RIAE analysis [29] of the LA-catalyzed aziridine ring-opening of 2-phenyl-1-methanesulfonylaziridine (2PMA, **19**) in the presence of ketone **8** is performed to elucidate the electronic factors responsible for the feasibility of these formal 32CA reactions.

## 2. Results and Discussion

The present MEDT study has been organized into five sections: (i) the first section presents the electron localization function [30] (ELF) topological analyses of the ground state (GS) electronic structures of 2-phenylaziridine (2PA **20**), 2PTA **4**, and the 2PTA:BF_3_ complex **21**; (ii) the second section encompasses an analysis of the DFT-based reactivity indices [31,32] calculated at the GS of the reagents; (iii) the third section analyzes the non-catalyzed aziridine ring-opening in 2PTA **4** by ketone **8**; (iv) the four section describes the reaction paths associated with the BF_3_ LA-promoted formal 32CA reaction of 2PTA **4** with ketone **8**; and (v) the final section presents a RIAE analysis [29] of the aziridine ring-opening of 2PMA **19** in the presence of ketone **8**, aimed at elucidating the electronic effects of the LAs on the aziridine ring-opening processes.

**Scheme 7:**
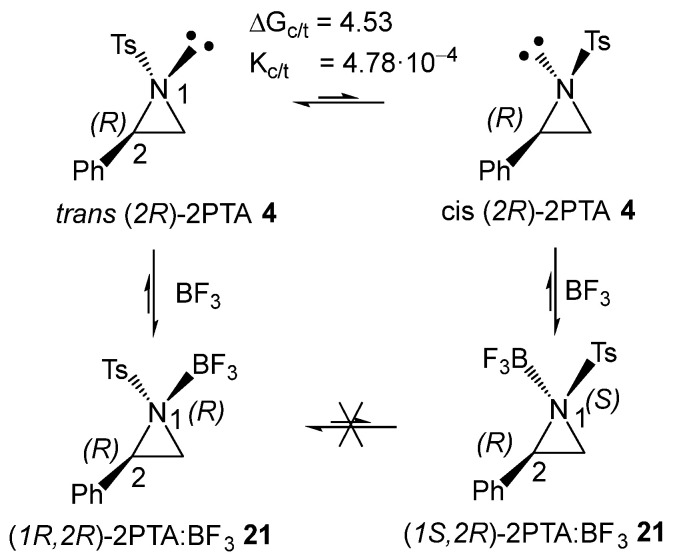
*trans* and *cis* conformations of (2*R*)-2PTA **4**, and the (1*R*,2*R*) and (1*S*,2*R*) diastereomers of the 2PTA:BF_3_ complex **21**. Relative Gibbs free energies, computed at 25 °C and 1 atm in DCM, are given in kcal·mol^−1^.

The (2*R*) and (2*S*) enantiomers of 2PTA **4** can be found in two conformations, in which the tosyl N1 substituent adopts a *trans* or *cis* relationship with the phenyl C2 substituent with respect to the aziridine ring plane (see the *trans* and *cis* conformations of (2*R*)-2PTA **4** in Scheme 7). Full optimization of the *trans* and *cis* conformations of (2*R*)-2PTA **4**, followed by thermodynamic calculations, indicates that the *cis* conformation is 4.53 kcal·mol^−1^ higher in Gibbs free energy than the *trans* one, due to steric repulsions between the two substituents in the *cis* configuration (see Figure S1 in the Supplementary Material). This Gibbs free energy difference yields a *cis*/*trans* ratio of 4.78·10^−4^, indicating that the *cis* conformer is chemically irrelevant under reaction conditions. Therefore, the regioisomeric reaction paths associated with the LA-catalyzed formal 32CA reaction were analyzed starting from the in situ generated (1*R*,2*R*)-2PTA:BF_3_ complex **21**.

### 2.1. ELF Analysis of the Electronic Structures of 2PA ***20***, 2PTA ***4***, and the 2PTA:BF_3_ Complex ***21***

**Figure 1:**
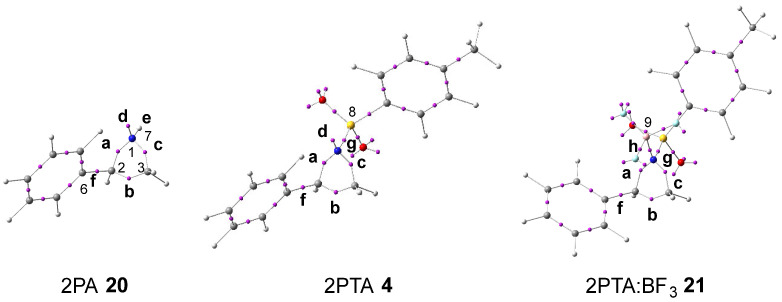
*ω*B97X-D/6-311G(d,p) ELF basin attractor positions, at 2PA **20**, 2PTA **4**, and the 2PTA:BF_3_ complex **21** in DCM. Letters **a**–**h** refer to the most relevant valence basins.

The topological analysis of the ELF [30] provides a quantitative description of the electron-density distribution within a molecule [33], thereby establishing a direct relationship between its electronic structure and reactivity. Consequently, the ELF topological analysis of aziridine 2PA **20**, 2PTA **4**, and the 2PTA:BF_3_ complex **21** was carried out in DCM. The spatial distribution of the ELF basin attractors is depicted in Figure 1. The populations of the most significant valence basins **a**–**h** are summarized in Table 1.

Examination of the ELF for aziridine 2PA **20** reveals a V(C2,C3) disynaptic basin, **b**, integrating 1.83 e; a V(N1,C2) disynaptic basin, **a**, integrating 1.44 e; a V(N1,C3) disynaptic basin, **c**, integrating 1.46 e; a V(C2,C6) disynaptic basin, **f**, integrating 2.14 e; a V(N1,H7) disynaptic basin, **e**, integrating 2.08 e; and a V (N1) monosynaptic basin, **d**, integrating 2.49 e. The V(N1,C2) and V(N1,C3) disynaptic basins show depleted electron populations, consistent with the weakened single bonds resulting from the higher electronegativity of nitrogen relative to carbon. Hence, the V(N1) monosynaptic basin, corresponding to the nitrogen non-bonding electron density, integrates more than 2.00 e.

Incorporation of the electron-withdrawing (EW) tosylate group in 2PTA **4**, followed by the coordination of BF_3_ LA in the 2PTA:BF_3_ complex **21**, produces no significant modification in their electronic structures relative to that of 2PA **20** (see Table 1). The populations of the V(C2,C3) and V(C2,C6) disynaptic basins, **b** and **f**, remain practically constant, while those of the V(N1,C2) and V(N1,C3) disynaptic basins, **a** and **c**, exhibit a slight increase. The population of the V(N1,B9) disynaptic basin, **h**, formed upon complexation with BF_3_ LA in 2PTA:BF_3_ complex **21**, is only 0.21 e lower than that of the V(N1) monosynaptic basin, **d**, associated with the nitrogen non-bonding electron density in 2PTA **4**.

Based on the topological analysis of the ELF of 2PA **20**, 2PTA **4**, and the 2PTA:BF_3_ complex **21**, it is clear that neither the presence of the EW tosyl substitution in 2PTA **4** nor the formation of the LA complex 2PTA:BF_3_ **21** originates any fundamental change in the GS electronic structure of the aziridine ring of 2PA **20**.

### 2.2. Analysis of the DFT-Based Reactivity Indices at the GS of the Reagents

The DFT-based reactivity indices [31,32] evaluated at the GS of the reagents provide an effective framework for rationalizing reactivity trends in polar reactions [32]. In particular, electrophilicity [34] ω and nucleophilicity [35] *N* indices allow prediction of the GEDT [25] occurring at the TSs. All reactivity indices were calculated at the B3LYP/6-31G(d) level, consistent with the theoretical definitions of the electrophilicity and nucleophilicity scales [32]. The global reactivity indices, electronic chemical potential *μ*, chemical hardness *η*, electrophilicity ω, and nucleophilicity *N* for aziridine **22**, its derivatives **4**, **20**, and **21**, and the ketone **8** are reported in Table 2.

The simplest aziridine **22** has electrophilicity [34] ω and nucleophilicity [35] *N* indices of 0.28 and 2.49 eV, respectively, and is classified as a marginal electrophile and a moderate nucleophile [32]. 2PA **20** exhibits electrophilicity ω and nucleophilicity *N* indices of 0.74 and 2.91 eV, respectively, classified as a moderate electrophile and on the borderline of a strong nucleophile. The presence of the phenyl substituent at the C2 carbon of 2PA **20** centarslightly increases the electrophilicity of the simplest aziridine **22**. Still, this increase is insufficient to allow for an SN reaction with nucleophiles.

2PTA **4** exhibits electrophilicity ω and nucleophilicity *N* indices of 1.33 and 2.06 eV, respectively, classifying it as a strong electrophile and a moderate nucleophile. Upon coordination of the LA BF_3_ to the N1 nitrogen atom of 2PTA **4**, the electrophilicity of 2PTA:BF_3_ **21** increases markedly to 1.73 eV, while its nucleophilicity decreases to 1.64 eV.

The electrophilicity ω and nucleophilicity *N* indices of ketone **8** are 0.95 and 2.48 eV, respectively, classifying it as a moderate electrophile and a moderate nucleophile. Accordingly, ketone **8** is expected to behave as a nucleophile when reacting with strong electrophiles displaying ω values greater than 2.0 eV [36].

Nucleofugality describes the ability of an atom or a group of atoms to leave with the bonding electron pair during a heterolytic bond dissociation [37]. This property defines the effectiveness of the LG in SN processes. The nucleofugality Λ index [26,28], formally derived from the electrophilicity index *ω* of the CH_3_–LG species, yet conceptually distinct from it, is a quantitative measure of the LG ability that governs SN reactions [23,24]. The nucleofugality Λ indices computed for methylamine **29**, sulfonamides **25** and **28**, and their LA complexes **23**, **24**, and **26** are collected in Table 3.

As shown in Table 3, the nucleofugality Λ values of the simplest aziridine **22** (see its electrophilicity w index in Table 2) and methylamine Me–NH_2_ **29** are 0.28 and 0.23 eV, respectively, and are significantly lower than that of methyl bromide, Me–Br **27**, Λ = 0.90 eV, used here as the reference [28]. These nucleofugality Λ values indicate that the amines –NH_2_ are markedly poorer LGs than bromide –Br. Consequently, such species are not expected to undergo SN reactions owing to the very low nucleofugality of the amine –NH_2_ group [26,28]. *N*-methyl-methanesulfonamide **28**, shows a nucleofugality Λ value of 0.48 eV, indicating improved but still limited LG ability relative to –Br.

In the series of BH_3_ and BF_3_ LA complexes, the nucleofugality Λ index of the corresponding Me–NHMes:LA species increases with the acidity of the LA, from 1.08 (**26**) to 1.32 (**24**) eV, indicating enhanced LG ability of the NHMes:LA moiety. Notably, the NHMes:BF_3_ group is a better LG than bromide.

Finally, *N*-methyl-toluenesulfonamide **25** and *N*-methyl-toluenesulfonamide/BF_3_ complex **23** exhibit nucleofugality Λ indices of 1.29 and 1.79 eV, respectively, making them more effective LG than the corresponding mesylates. The 0.50 eV increase in the Λ value upon BF_3_ coordination highlights the catalytic effect of the LA. Notably, the –NHTos:BF_3_ complex is the most efficient LG in this series.

### 2.3. Study of the Non-Catalyzed Aziridine Ring-Opening in 2PTA ***4*** by Ketone ***8***

**Scheme 8:**
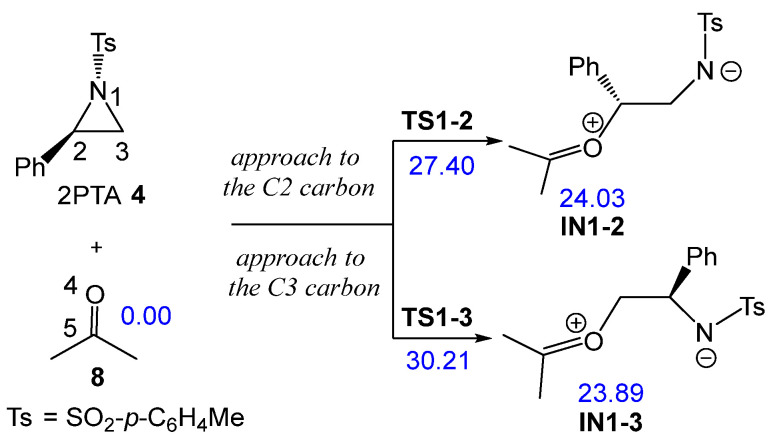
Regioisomeric reaction paths associated with the aziridine ring-opening in 2PTA **4** by ketone **8**. *ω*B97X-D/6-311G(d,p) relative enthalpies in DCM, in kcal·mol^−1^, are given in blue.

The non-catalyzed aziridine ring-opening in (*2R*)-2PTA **4** by ketone **8** was first studied. Because the phenyl group is attached to the C2 carbon of the aziridine ring, there are two possible regioisomeric pathways for opening the aziridine ring. These involve the approach of the O4 oxygen of ketone **8** to either C2 or C3 carbons of the aziridine ring in 2PTA **4** (see Scheme 8). The *ω*B97X-D/6-311G(d,p) relative enthalpies in DCM of the stationary points involved in the ring-opening step are shown in Scheme 8, whereas the thermodynamic data are listed in Table S1 in the Supplementary Material.

The reaction between aziridine 2PTA **4** and ketone **8** proceeds through two possible reaction paths involving the approach of ketone **8** at the C2 or C3 carbon of the aziridine ring. The corresponding TSs, **TS1-2** and **TS1-3**, present activation enthalpies of 27.40 and 32.21 kcal·mol^−1^, respectively. Formation of the zwitterionic intermediates **IN1-2** and **IN1-3** is markedly endothermic, by 24.03 and 23.89 kcal·mol^−1^, respectively. These results indicate that the non-catalyzed ring-opening of 2PTA **4** by ketone **8** is energetically unfavorable from both kinetic and thermodynamic standpoints. The lower energy of **TS1-2**, which is 2.81 kcal·mol^−1^ below **TS1-3**, confirms a clear preference for the C2-regioisomeric pathway.

**Figure 2:**
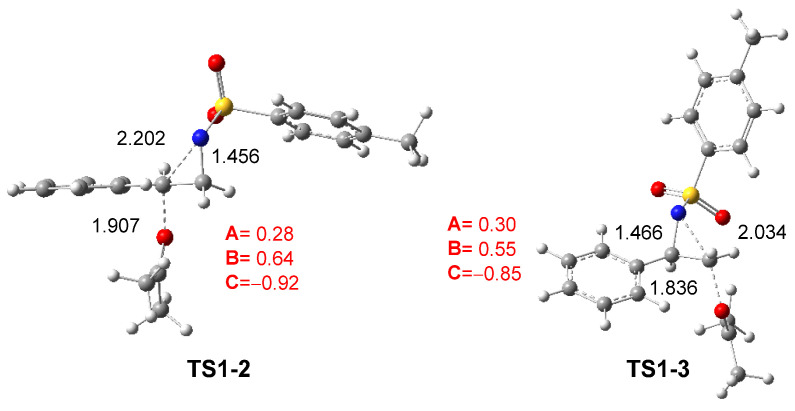
*ω*B97X-D/6-311G(d,p) optimized geometries in DCM of **TS1-2** and **TS1-3**. The distances are given in Angstroms. The sum of the natural charges of the atoms belonging to the **A**, **B**, and **C** frameworks of **TS1-2** and **TS1-3**, in average of the electrons e, is given in red.

The geometries of **TS1-2** and **TS1-3** optimized in DCM are depicted in Figure 2. The C2–N1 and C3–N1 distances at these TSs are 2.202 and 2.034 Å, respectively, while the O4–C2 and O4–C3 distances between the oxygen of ketone **8** and the two aziridine carbons are 1.907 and 1.836 Å. At the preferred **TS1-2**, the opening of the aziridine ring is more advanced, while the approach of ketone **8** is more delayed. As shown in Scheme 8 and Figure 2, the approach of ketone **8** to the chiral C2 carbon of *trans* (2*R*)-2PTA **4** leads to an inversion of the configuration at the aziridine C2 carbon.

The GEDT [25] was computed at **TS1-2** and **TS1-3** in DCM to examine the changes in the electron density at these TSs. For this purpose, the atomic charges at the TSs were grouped into three frameworks: the ketone framework **A**, the ethylene framework **B**, and the sulfonamide framework **C**. The sums of the natural charges [38,39] for each framework are reported in Figure 2. The sulfonamide framework **C** exhibits notable negative charges by −0.92 and −0.85 e at **TS1-2** and **TS1-3**, respectively, while the phenylethylene framework **B** is positively charged by 0.64 and 0.55 e. These data, analogous to those found in SN processes [23,24], suggest that the N1–C2 single bond in aziridine 2PTA **1** is already broken. This charge analysis also reveals the participation of the ketone **A** and sulfonamide **C** frameworks in stabilizing the central cationic phenylethylene framework **B** at the more favorable **TS1-2** [28].

**Figure 3:**
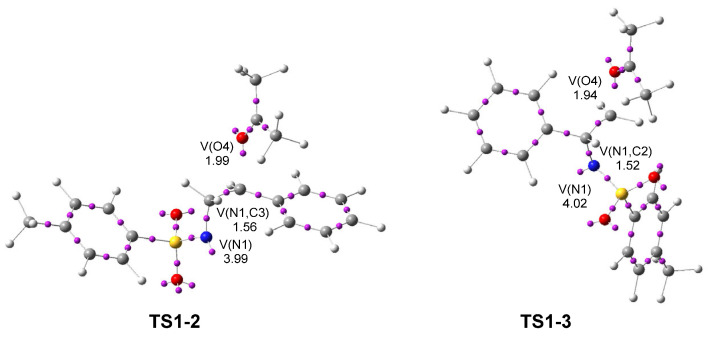
*ω*B97X-D/6-311G(d,p) ELF basin attractor positions, at **TS1-2** and **TS1-3**. The populations of the more relevant valence basins are given in average number of electrons, e.

Next, a topological analysis of the ELF of **TS1-2** and **TS1-3** was performed (see Figure 3). Both TSs show a V(N1) monosynaptic basin, integrating 3.99 and 4.02 e, respectively, associated with non-bonding electron density at the N1 nitrogen. They also display a V(N1,C3) disynaptic basin integrating 1.56 e at **TS1-2**, and a V(N1,C2) disynaptic basin integrating 1.52 e at **TS1-3**. The population of the V(N1) monosynaptic basin at 2PTA **1** was 3.56 e, indicating an increase of about 0.5 e after the heterolytic breaking of the N1–C2[3] single bonds.

The ketone framework shows a V(O4) monosynaptic basin integrating 1.99 e (**TS1-2**) and 1.94 e (**TS1-3**) in the direction to the C2[3] carbon, which are associated with non-bonding electron density on the ketone O4 oxygen. The absence of any V(N1,C2) or V(N1,C3) disynaptic basins at the two TSs indicates that the aziridine N1–C2 and N1–C3 single bonds have already been broken at **TS1-2** and **TS1-3**, respectively. Likewise, the absence of a V(C2[3],O4) disynaptic basin reveals that the formation of the new C2[3]–O4 single bonds has not yet begun.

### 2.4. BF_3_ LA-Promoted Formal 32CA Reaction of 2PTA **4** with Ketone **8**

**Scheme 9:**
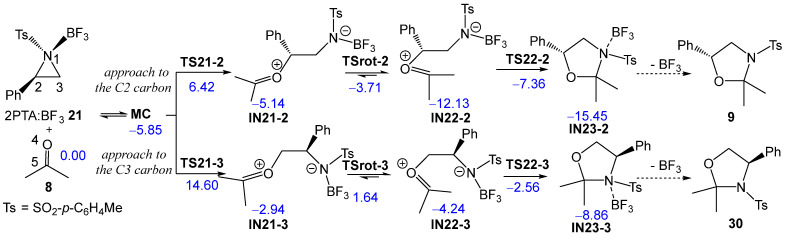
Regioisomeric reaction paths associated with the formal 32CA reaction of 2PTA:BF_3_ complex **21** with ketone **8**. *ω*B97X-D/6-311G(d,p) relative enthalpies in DCM, in kcal·mol^−1^, are given in blue.

Next, the two regioisomeric reaction paths associated with BF_3_ LA-promoted formal 32CA reaction of (2*R*)-2PTA **4** with ketone **8**, yielding 1,3-oxazolidine **9**, were studied (see Scheme 9). The BF_3_ LA-promoted formal 32CA reaction occurs via a stepwise mechanism. The two main steps of this stepwise mechanism are: (i) the aziridine ring-opening of the (1*R*,2*R*)-2PTA:BF_3_ complex **21** in the presence of ketone **8**, yielding the zwitterionic intermediates **IN21-2** and **IN21-3**; and (ii) the final ring closure at the conformational zwitterionic intermediates **IN22-2** and **IN22-3**, results in the formation of the BF_3_ coordinated 1,3-oxazolidine intermediates **IN23-2** and **IN23-3**. The aziridine ring-opening of the (1*R*,2*R*)-2PTA:BF_3_ complex **21** via the regioisomeric **TS21-2** or **TS21-3** results in the loss of the chiral character of the aziridine N1 nitrogen atom. Consequently, the resulting zwitterionic intermediates **IN-21-2** and **IN-21-3** lose configurational identity at N1 and can interconvert, giving rise to the conformational intermediates **IN22-2** and **IN22-3**, respectively. The *ω*B97X-D/6-311G(d,p) relative enthalpies in DCM are given in Scheme 9, while the thermodynamic data are given in Table S2 in the Supplementary Material.

During the approach of ketone **8** to the 2PTA:BF_3_ complex **21**, a weak molecular complex (MC) was identified and characterized. **MC** lies 5.85 kcal·mol^−1^ below the energy of the separated reagents, indicating that its formation is not significant in the reaction pathways since its formation is endergonic by 4.40 kcal·mol^−1^ (see below). The optimized geometry of **MC** is shown in Figure S2 in the Supplementary Material. At MC, ketone **8** is positioned parallel to approximately 3.0 Å of the 2PTA:BF_3_ complex **21**.

The activation enthalpy associated with the nucleophilic approach of the O4 oxygen of ketone **8** to the C2 and C3 carbons of the aziridine ring in the 2PTA:BF_3_ complex **21** along the N1–C2 and N1–C3 breaking bonds, via **TS21-2** and **TS21-3**, is 6.42 and 14.60 kcal·mol^−1^, respectively; the formation of intermediates **IN21-2** and **IN21-3** is exothermic by −5.14 and −2.94 kcal·mol^−1^. The zwitterionic intermediates **IN21-2** and **IN21-3** are in equilibrium with the rotamers **IN22-2** and **IN22-3**, which, by an intramolecular nucleophilic attack of the N1 nitrogen on the C5 carbon of the ketone framework, via **TS22-2** and **TS22-3**, respectively, afford the BF_3_-coordinated 1,3-oxazolidine intermediates **IN23-2** and **IN23-3**. The TSs associated with the C2–C3 single bond rotation, demanded for the conversion of **IN21-2** and **IN21-3** into **IN22-2** and **IN22-3**, respectively, lie 1.41 (**TSrot-2**) and 4.59 (**TSrot-3**) kcal·mol^−1^ above their corresponding intermediates. From **IN22-2** and **IN22-3**, the activation enthalpies associated with **TS22-2** and **TS22-3** are 4.77 and 1.68 kcal·mol^−1^, respectively; formation of the BF_3_-coordinated 1,3-oxazolidines **IN23-2** and **IN23-3** is exothermic by −15.45 and −8.86 kcal·mol^−1^. The activation energies associated with the formation of the oxazolidine ring via **TS22-2** and **TS22-3** are lower than those associated with the LA-catalyzed opening of the aziridine ring via **TS21-2** and **TS21-3**, indicating that the latter represents the rate-determining step of this stepwise mechanism. The aziridine ring-opening of the 2PTA:BF_3_ complex **21** in the presence of ketone **8** is completely C2 regioselective as **TS21-3** is 8.18 kcal·mol^−1^ higher in enthalpy than **TS21-2**.

The stereoisomeric **TS21-2’** and **TS21-3’** associated with the ketone promoted aziridine ring-opening of the diastereomeric (1*S*,2*R*)-2PTA:BF_3_ complex **21** were also studied. The corresponding thermodynamic data are given in Table S2, while the TS geometries are given in Figure S3 in the Supplementary Material. These diastereomeric TSs are 1.00 and 0.11 kcal·mol^−1^ higher in enthalpy than their (1*R*,2*R*) counterparts, **TS21-2** and **TS21-3**, respectively. After passing **TS21-2’** and **TS21-3’**, the resulting intermediates are conformers of those obtained via **TS21-2** and **TS21-3** as a consequence of the loss of the chiral character of the aziridine N1 nitrogen atom.

**Figure 4:**
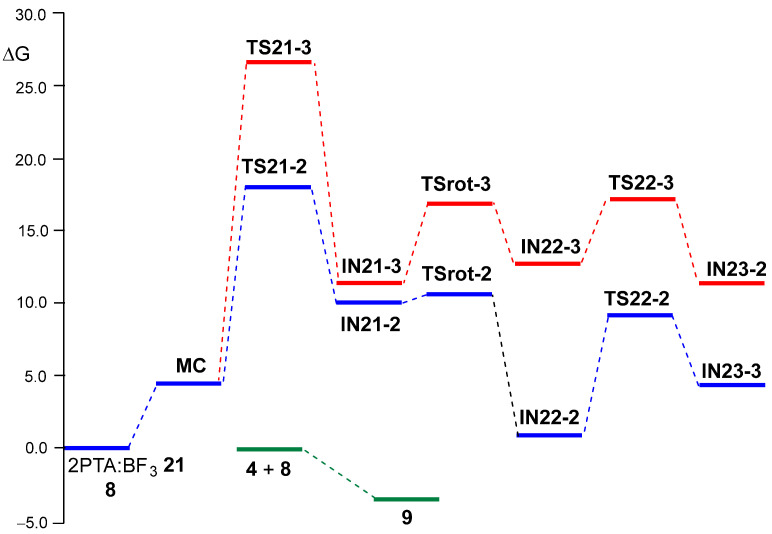
*ω*B97X-D/6-311G(d,p) Gibbs free energy profiles, ΔG in kcal·mol^–1^, computed at 25 °C and 1 atm in DCM, for the two regioisomeric reaction paths associated with BF_3_ LA-promoted formal 32CA reaction of 2PTA:BF_3_ complex **21** with ketone **8**. The more favorable C2 regioisomeric reaction path is given in blue, while the C3 one is in red. The reaction Gibbs free energy of the formal 32CA reaction of 2PTA **4** with ketone **8**, yielding 1,3-oxazolidine **9**, is given in green.

A representation of the Gibbs free energy profiles associated with the two regioisomeric reaction paths of the BF_3_ LA-promoted formal 32CA reaction of 2PTA:BF_3_ complex **21** with ketone **8** is shown in Figure 4. Table 4 summarizes the relative enthalpies, entropies, and Gibbs free energies of all stationary points, while the complete thermodynamic data are provided in Table S2 in the Supplementary Material. Figure 4 also displays the reaction Gibbs free energy of the overall formal 32CA reaction. The corresponding thermodynamic data are provided in Table S3.

Several important conclusions can be drawn from the relative Gibbs free energies given in Table 4: (i) the relative Gibbs free energies, ranking from 10 to 20 kcal·mol^–1^, are higher than the corresponding relative enthalpies as a consequence of the unfavorable activation entropies associated with this bimolecular processes, between −34 and −68 cal·mol^–1^K^–1^; (ii) with the formation of **MC**, the TSs and intermediates are endergonic, and all stationary points associated with the regioisomeric C3 reaction path are located above those associated with the C2 one (see Figure 4); (iii) the activation Gibbs free energy associated with aziridine ring-opening via **TS21-2**, 18.00 kcal·mol^–1^, is higher than that associated with the ring-closure of **IN22-2** via **TS22-2**, 9.18 kcal·mol^–1^, and consequently, the aziridine ring-opening constitutes the rate-determining step of this BF_3_ LA-promoted formal 32CA reaction; (iv) this reaction is completely C2 regioselective, as **TS-21-3** is 8.68 kcal·mol^−1^ higher in Gibbs free energy than **TS21-2**, in complete agreement with the experimental outcomes [19]; (v) **TSrot-2** is located 7.88 kcal·mol^–1^ below **TS21-2**, and consequently, after passing **TS21-2**, the reaction continues to form the BF_3_ coordinated 1,3-oxazolidine **IN23-2**; and finally, (vi) the formal 32CA reaction of 2PTA **4** with ketone **8** is exergonic by 3.60 kcal·mol^–1^, leading to the complete formation of 1,3-oxazolidine **9** in complete agreement with the experiment outcomes [19] (see Figure 4). Note that this formal 32CA reaction is exothermic by 19.73 kcal·mol^–1^; the unfavorable activation entropy associated with this bimolecular process, −54.08 cal·mol^−1^K^−1^, reduces the exergonic character of the reaction (see Table 4).

**Figure 5:**
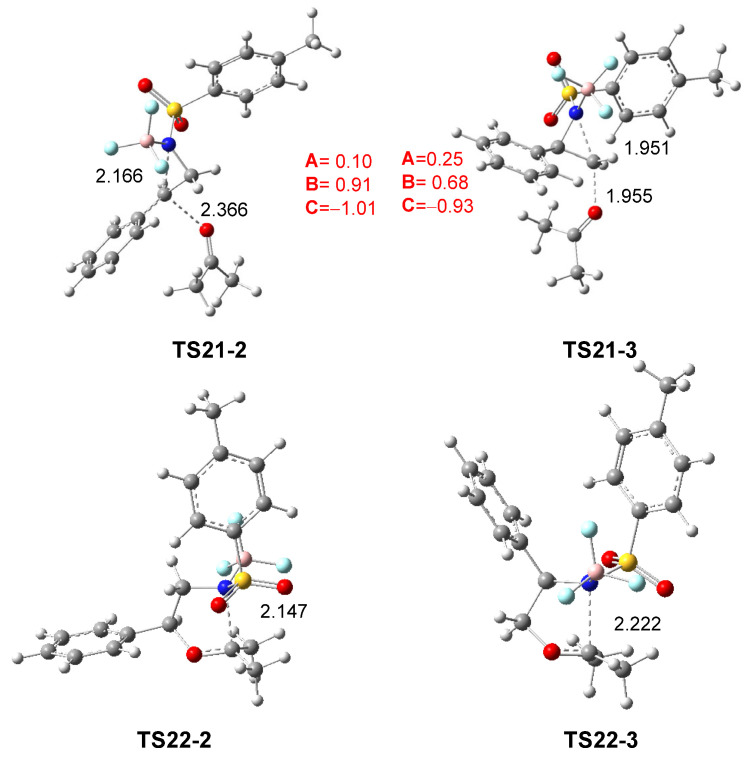
*ω*B97X-D/6-311G(d,p) optimized geometries in DCM of **TS21-2**, **TS21-3**, **TS22-2** and **TS22-3**. The distances are given in Angstroms. The sum of the natural charges of the atoms belonging to the **A**, **B**, and **C** frameworks of **TS21-2** and **TS21-3**, in average of the electrons e, is given in red.

The geometries of **TS21-2** and **TS21-3**, associated with the aziridine ring-opening of 2PTA:BF_3_ complex **21** in the presence of ketone **8**, and those of **TS22-2** and **TS22-3** associated with the ring-closure in the zwitterionic intermediates **IN22-2** and **IN22-3**, in DCM, are shown in Figure 5. The distances between the C2 and C3 carbons and the N1 nitrogen in the first two TSs are 2.166 and 1.951 Å, respectively, while the distances between the ketone O4 oxygen and these carbons are 2.366 and 1.955 Å. In the most favorable **TS21-2**, while the opening of the aziridine ring is more advanced, the approach of ketone **8** is clearly more delayed. The C3–C2–Ph angle at **TS21-2** is 127.6 degrees, reflecting the trigonal planar structure of the C2 carbon. At the TSs associated with the ring-closure, **TS22-2** and **TS22-3**, the N1–C5 distance is 2.147 and 2.222 Å, respectively.

As shown in Scheme 9 and Figure 5, the approach of ketone **8** to the chiral C2 carbon of (1*R*,2*R*)-2PTA:BF_3_ complex **21** leads to an inversion of the configuration at the aziridine C2 carbon of the complex **21** relative to that present in the final oxazolidine **9** (see the experimental LA-catalyzed formal 32CA reaction shown in Scheme 3 and Scheme 4). This stereochemical outcome is classically associated with an S_N_2-type mechanism for aziridine ring-opening [19].

The geometries of the stereoisomeric **TS21-2’** and **TS21-3’** associated with the ketone promoted aziridine ring-opening of the (1*S*,2*R*)-2PTA:BF_3_ complex **21** are shown in Figure S3 in the Supplementary Material. As expected, a comparison of the distances between the C2 and C3 carbons and the N1 nitrogen at the two pairs of diastereomeric (1*R*,2*R*) and (1*S*,2*R*) TSs reveals no significant differences.

A comparison of the energetic and geometrical parameters of the diastereomeric (1*R*,2*R*) and (1*S*,2*R*) TSs indicates that the change in the N1 configuration of 2PTA:BF_3_ complex **21** does not cause significant changes in the mechanism of the LA-catalyzed aziridine ring-opening of 2PTA **4**.

The GEDT was computed at **TS21-2** and **TS21-3** associated with the aziridine ring-opening step in DCM. The atoms of the two TSs were divided into three frameworks: the ketone **A**, the phenylethylene moiety **B**, and the LA-coordinated sulfonamide group **C**. The sums of the natural charges for each framework at the two TSs are shown in Figure 5. While the sulfonamide:BF_3_ framework **C** is charged by −1.01 and −0.93 e at **TS21-2** and **TS21-3**, respectively, the phenylethylene framework **B** is positively charged by 0.91 and 0.68 e. The presence of the LA catalyst, coordinated to sulfonamide, increases the separation of charges between the **B** and **C** frameworks. At the most favorable **TS21-2**, the charges of the **B** and **C** frameworks are higher than those at **TS21-3**, whereas the charge of the ketone **A** framework is lower by 0.10 and 0.25 e, respectively. The charge analysis at **TS21-2**, which is similar to those of the TSs associated with an S_N_1-like mechanism [24,28], and those found at the TS associated with the BF_3_ LA-catalyzed C2 regioselective aziridine ring-opening process of 2PNSA **16** by chloride anion Cl^−^ [28] (see Scheme 6), together with the trigonal planar structure of the C2 carbon, suggest that the LA-promoted aziridine ring-opening process at the 2PTA:BF_3_ complex **21** takes place via a S_N_1-like mechanism [28].

**Figure 6:**
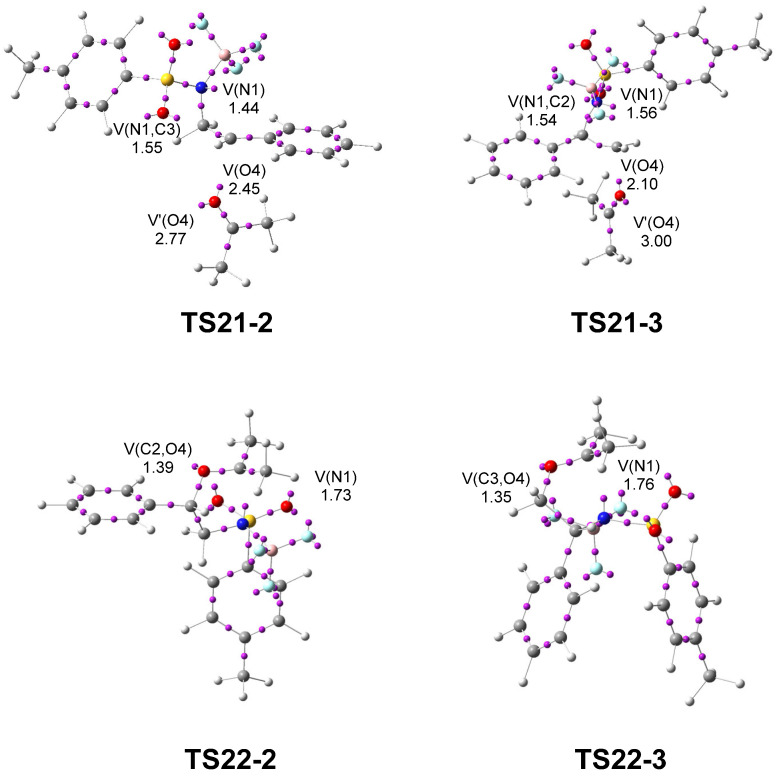
*ω*B97X-D/6-311G(d,p) ELF basin attractor positions, at **TS31-2** and **TS31-3**. The populations of the valence basins are given in average number of electrons, e.

Next, a topological analysis of the ELF was performed for **TS21-2** and **TS21-3**, associated with the regioisomeric aziridine ring-opening processes, and for **TS22-2** and **TS22-3**, corresponding to the ring-closure leading to the formation of the oxazolidine ring (see Figure 6).

The ELF analyses of **TS21-2** and **TS21-3** show the presence of a V(N1) monosynaptic basin, integrating 1.44 and 1.56 e, respectively, associated with non-bonding electron density on the N1 nitrogen, as well as a V(O4) monosynaptic basin, integrating 2.45 e and 2.10 e along the O4–C2 direction, associated with non-bonding electron density on the O4 oxygen. The presence of the V(N1) monosynaptic basins at the two TSs, which are absent in 2PTA:BF_3_ complex **21** (see Figure 1), indicates that the N1–C2[3] single bonds have already been broken at these TSs. In contrast, the absence of a V(C2[3],O4) disynaptic basin indicates that the formation of the new C2[3]–O4 single bonds has not yet begun. The populations of the V(N1) monosynaptic basins and that of the V(O4) monosynaptic basin at both TSs indicate that the most favorable **TS21-2** is slightly more delayed. The combined ELF and GEDT analyses at the most favorable **TS21-2** clearly indicate that this TS corresponds to an S_N_1-like mechanism [24,28].

ELF of **TS22-2** and **TS22-3** shows the presence of a V(N1) monosynaptic basin, integrating 1.73 and 1.76 e, respectively, associated with a non-bonding electron density at the N1 nitrogen, and a V(C2[3],O4) disynaptic basin, integrating 1.39 and 1.35 e, respectively, associated with the new C2[3]–O4 single bonds created in the first step of this stepwise formal 32CA reaction. On the other hand, the absence of a V(N1,C5) disynaptic basin indicates that the formation of the new N1–C5 single bonds has not yet begun.

### 2.5. RIAE Analysis of the Role of the BF_3_ LA-Catalyst in the Aziridine Ring-Opening Step

**Scheme 10:**
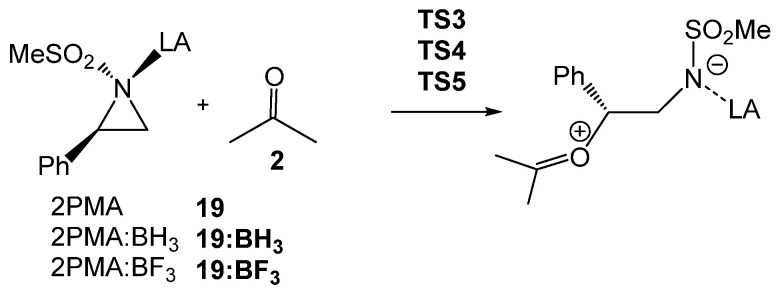
Aziridine ring-opening of 2PMA **19** in the presence of ketone **8**, as well as in the absence of and in the presence of the BH_3_ and BF_3_ LAs.

Finally, a RIAE analysis [29] was performed for the aziridine ring-opening step of 2PMA **19** in presence of ketone **8**, and in the absence and presence of the two LAs of increasing acidity, BH_3_ < BF_3_, in order to elucidate how the electronic effects of the BF_3_ LA influence the reduction in activation energies in the LA-catalyzed aziridine ring-opening processes (see Scheme 10). In the current RIAE analysis, the toluene group present in 2PTA **4** has been replaced by a methyl group in 2PMA **19**. This RIAE analysis was performed at the M06-2X/6-311G(d,p) computational level in the gas phase as required by the Interacting Quantum Atom (IQA) calculations [40]. The M06-2X/6-311G(d,p) gas phase total and relative energies of the reagents and TSs shown in Scheme 10 are given in Table S4 in the Supplementary Material.

The reagents and TSs involved in the C2 regioisomeric reaction paths were fully optimized in the gas phase at the M06-2X/6-311G(d,p) computational level. As in the previous GEDT analyses, the atoms belonging to the reagents and TSs were regrouped into the ketone **A**, phenyl ethylene **B**, and sulfonamide **C** frameworks in this RIAE analysis. The M06-2X/6-311G(d,p) gas-phase ξ$E_{intra}^{X}$ intra-atomic, ξ$E_{inter}^{X}$ inter-atomic and ξ$E_{total}^{X}$ total energies of the **A**, **B**, and **C** frameworks at the TSs relative to their GSs are provided in Table 5 (see Computational Details). The sum of the M06-2X/6-311G(d,p) gas-phase natural changes in the **A**, **B**, and **C** frameworks is provided in Table S5 of the Supplementary Material.

Analysis of the standard deviation of the ξ$E_{total}^{X}$ total energies of the three frameworks indicate that the stabilization of the sulfonamide LG **C** framework caused by the presence of the LA, together with the decrease in the destabilization of the ketone **A** framework, are the main factors responsible for the overall decrease in the ξ$E_{total}^{A+B+C}$ total energies of the two LA-catalyzed aziridine ring-opening processes, which represent the RIAE activation energies. Thus, the negative stabilizing ξ$E_{total}^{C}$ total energies of the sulfonamide **C** framework increase in the order −3.66 (**TS3**) > −12.01 (**TS4**) > −19.08 (**TS5**) kcal·mol^−1^. Note that at the three TSs, the ξ$E_{total}^{C}$ total energies of the sulfonamide **C** framework are the only stabilizing factor. On the other hand, the destabilizing ξ$E_{total}^{A}$ total energies of the ketone **A** framework decrease in the same order 27.69 (**TS3**) > 23.84 (**TS4**) > 17.84 (**TS5**) kcal·mol^−1^.

An analysis of the factors governing the stabilization of the sulfonamide **C** framework indicates that the increase in the stabilizing ξ$E_{inter}^{C}$ inter-atomic contribution plays a key role in the LA-catalyzed processes, leading to lower activation energies (see Table S4 in the Supplementary Material). This effect is consistent with the elongation of the N1–C2 bond at **TS21-2** (see Figure 5), reflecting an enhanced heterolytic cleavage of this bond, as well as with the increase in negative charge accumulated on the sulfonamide framework upon LA coordination (see Figure 5).

On the other hand, a detailed analysis of the factors responsible for the decrease in the destabilization of the ketone **A** framework indicates that the decrease in the destabilizing ξ$E_{inter}^{A}$ inter-atomic energies play a decisive role. The decrease in ξ$E_{inter}^{A}$ inter-atomic energy observed upon LA coordination can be rationalized in terms of changes in the electronic structure of the reacting system. As revealed by the GEDT and RIAE analyses, LA coordination alters the electron density response along the reaction coordinate, thereby reducing destabilizing inter-atomic interactions within the ketone framework. In particular, the interaction with the LA limits the extent of electron density polarization required to accommodate the reaction progress, resulting in lower repulsive inter-atomic energy components. Consequently, the stabilization of the ketone **A** framework arises from an electronically driven effect induced by LA coordination, consistent with the energetic trends reported in Table 5, rather than from purely geometric or steric factors.

**Figure 7:**
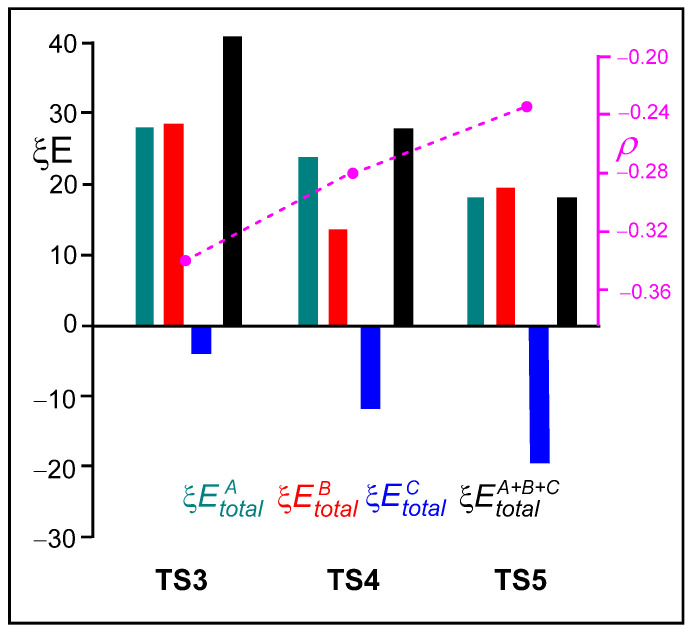
Graphical representation of the sets of the ξ $E_{total}^{A}$, ξ$E_{total}^{B}$, ξ$E_{total}^{C}$, and ξ$E_{total}^{A+B+C}$ total energies, given in kcal·mol^−1^, for the TSs associated with the non-catalyzed and LA-catalyzed aziridine ring-opening of 2PMA **19** in the presence of ketone **8**. The ξ$E_{total}^{A+B+C}$ total energies correspond to the RIAE relative energy of these reactions. ξ$E_{total}^{X}$ total energies of the **A**, **B**, and **C** frameworks at the four TSs are depicted in blue, red, and green, respectively. The black bar represents the ξ$E_{total}^{A+B+C}$ activation energies. The GEDT values at the sulfonyl aziridine framework, in average number of electrons e, are shown in pink.

Figure 7 presents a graphical representation of the ξ$E_{total}^{X}$ total energies of the **A**, **B**, and **C** frameworks at the three TSs, depicted in green, red, and blue, respectively. The ξ$E_{total}^{A+B+C}$ total energies, shown in black, correspond to the RIAE activation energies of these aziridine ring-opening processes. The GEDT values at the sulfonyl aziridine framework are shown in pink. Several important conclusions can be drawn from the graphical trends displayed in Figure 7: (i) The unfavorable ξ$E_{total}^{A}$ total energies associated with the ketone **A** framework decrease in the order **TS3** > **TS4** > **TS5**. In the same order, the positive charge on the ketone **A** framework, which acts as a nucleophile, also decreases. (ii) The presence of the LA markedly stabilizes the sulfonamide **C** framework. Accordingly, the favorable ξ$E_{total}^{C}$ total energies associated with the sulfonamide **C** framework increase in the opposite order, **TS3** < **TS4** < **TS5**, consistent with the increase in the negative charge on the sulfonamide **C** framework acting as the LG (see Table S5 in the Supplementary Material). (iii) The decrease in the GEDT from the ketone framework to the sulfonyl aziridine one with the increase in the acidic character of the LA results in the stabilization of the ketone framework, thus contributing to the decrease in the ξ$E_{total}^{A+B+C}$ total energies. Note that, in polar reactions, the nucleophiles are destabilized due to a loss of electron density. Finally, (iv) the decrease in the destabilization of the nucleophilic ketone **A** framework and the increase in the stabilization of the LG sulfonamide **C** framework, along with the analysis of the GEDT at these frameworks, suggests a shift in the mechanism of these ring-opening processes from an S_N_2-like to an S_N_1-like [28].

## 3. Computational Details

This MEDT study was conducted using quantum chemical calculations within Density Functional Theory [41] (DFT). The B3LYP [42,43], *ω*B97X-D [44] and M06-2X [45] functionals were used in combination with the standard 6-31G(d) and 6-311G(d,p) [46] basis sets. The choice of exchange–correlation functionals in this study follows the methodological requirements of the Molecular Electron Density Theory (MEDT) framework. The ωB97X-D functional was used to describe reaction profiles and TSs due to its reliable performance for thermochemistry, barrier heights, and long-range interactions relevant to LA complexes [44]. The M06-2X functional was employed in the RIAE analysis, as this approach is formulated within the IQA framework and implemented for this functional [29,45]. The mechanistic conclusions derived from the MEDT analysis rely on electronic structure trends that are independent of the specific functional used [22,23,24]. In the RIAE analyses [29], some stationary points were computed using the M06-2X functional. The TSs were characterized by the presence of only one imaginary frequency. Geometry optimizations were performed using the Berny method [47,48], and the intrinsic reaction coordinate [49] (IRC) calculations were performed to confirm the connection between each TS and its corresponding minima [50,51]. Solvent effects were taken into account by fully optimizing the gas-phase structures at the same computational level using the polarizable continuum model [52,53] (PCM) within the self-consistent reaction field formalism [54,55,56] (SCRF), considering DCM as solvent.

The GEDT [25] values were computed using the equation GEDT(f) = Σq_f_, where q are the natural charges [38,39] of the atoms belonging to one of the three frameworks (f) at the TS geometries. The DFT-based reactivity indices [31,32] were calculated according to equations reported in [32].

All quantum chemical calculations were performed using the Gaussian 16 software package [57]. ELF [30] analyses of the *ω*B97X-D/6-311G(d,p) monodeterminantal wavefunctions were carried out with the TopMod package [58], employing a cubical grid with a step size of 0.1 Bohr. Molecular geometries and ELF basin attractors were visualized by using the GaussView program [59].

The IQA [40] used in the RIAE analysis [29,60] was performed with the AIMAll package [61] using the corresponding M06-2X/6-311G(d,p) monodeterminantal pseudo-wavefunctions. It is worth noting that the AIMAll program allows the use of only the B3LYP and M06-2X functionals.

## 4. Conclusions

The LA-promoted formal 32CA reaction of 2PTA **4** with ketone **8**, yielding 1,3-oxazolidine **9**, experimentally studied by Ghorai and Ghosh [19], has been studied within the framework of MEDT at the *ω*B97X-D/6-311G(d,p) computational level in DCM. Topological analysis of the ELF of 2PA **20**, 2PTA **4**, and the 2PTA:BF_3_ complex **21** indicates that neither the presence of the EW tosyl substituent in 2PTA **4** nor the formation of the LA 2PTA:BF_3_ complex **21** originates a meaningful modification in the GS electronic structure of the aziridine ring of 2PA **20**. Analysis of the DFT-based reactivity indices indicates that although the formation of the 2PTA:BF_3_ complex **21** increases the electrophilicity of 2PTA **4**, the moderate nucleophilic character of ketone **8** does not favor a polar process. On the other hand, analysis of the nucleofugality Λ indices indicates that while the –NH_2_ group is a poor LG, the –NHTos and the –NHTos:BF_3_ groups are good LGs, favoring the aziridine ring-opening.

The non-catalyzed aziridine ring-opening of 2PTA **4** in the presence of ketone **8** has a very high activation enthalpy of 27.40 kcal·mol^−1^ via **TS1-2**; formation of the corresponding zwitterionic intermediates **IN1-2** is markedly endothermic by 24.03 kcal·mol^−1^. Thus, the non-catalyzed aziridine ring-opening is both kinetically and thermodynamically very unfavorable. Topological analysis of the ELF of **TS1-2** shows that while the C2–N1 single bond has already been broken, the formation of the new O4–C2 single bond has not begun at this TS.

The BF_3_ LA-promoted formal 32CA reaction of 2PTA **4** with ketone **8** takes place through a stepwise mechanism involving an initial BF_3_ LA-promoted aziridine ring-opening process, followed by a ring-closure process forming a 1,3-oxazolidine ring. The presence of the phenyl substituent at the C2 carbon of 2PTA **4** enables two competitive regioisomeric reaction paths, yielding two isomeric 1,3-oxazolidines: **9** and **30**. The activation enthalpy associated with the more favorable C2–N1 breaking bond via **TS21-2**, ΔH^≠^ = 6.42 kcal·mol^−1^, is 20.98 kcal·mol^−1^ lower in enthalpy than that for the non-catalyzed process via **TS1-2**, asserting the relevant role of the LAs. On the other hand, the C3–N1 breaking bond via **TS21-3**, ΔH^≠^ = 14.60 kcal·mol^−1^, is 8.18 kcal·mol^−1^ higher in enthalpy than **TS21-2**, causing the aziridine ring-opening process to be completely C2 regioselective. **TS22-2**, associated with the ring-closure process, presents an activation enthalpy of 4.77 kcal·mol^−1^. Analysis of the Gibbs free energy profile clearly indicates that the first step of the stepwise mechanism, which involves the LA-promoted aziridine ring-opening process, is the rate-determining step of the overall formal 32CA reaction.

Topological analysis of the ELF of the more favorable **TS21-2** indicates that the C2–N1 single bond has already broken, while the formation of the C2–O4 single bond has not yet begun. The trigonal planar character of the C2 carbon at **TS21-2**, together with the high positive charge of the phenyl ethylene framework, 0.91 e, suggests that the LA-promoted aziridine ring-opening of 2PTA **4** takes place via an S_N_1-like mechanism rather than an S_N_2-type, as proposed in the literature [19]. The proposed S_N_1-like mechanism accounts for the stereospecific character of this formal 32CA reaction, which proceeds with inversion of the aziridine C2 carbon.

A RIAE analysis of the non-catalyzed, and BH_3_ and BF_3_ LA-catalyzed aziridine ring-opening of 2PMA **19** in the presence of ketone **8** shows that the stabilization of the sulfonamide/LA LG and that of the ketone **8** frameworks are the main factors controlling the reduction in the activation energies in the LA-promoted processes. The decrease in destabilization of the nucleophilic ketone **A** framework and the increase in stabilization of the LG sulfonamide **C** framework, along with the analysis of the GEDT at these frameworks, suggest that the presence of LA BF_3_ shifts the mechanism of the ring-opening processes from an S_N_2-like at the non-catalyzed to an S_N_1-like at the BF_3_ LA-catalyzed process.

## Figures and Tables

**Table 1:** Populations, in average number of electrons, e, of the more relevant valence basins of 2PA **20**, 2PTA **4**, and the 2PTA:BF_3_ complex **21**. See Figure 1 for the assignment of the **a**–**h** valence basins.

		20	4	21
V(N1,C2)	**a**	1.44	1.49	1.59
V(C2,C3)	**b**	1.83	1.82	1.89
V(N1,C3)	**c**	1.46	1.51	1.67
V(N1)	**d**	2.49	2.56	
V(N1,H7)	**e**	2.08		
V(C2,C6)	**f**	2.14	2.13	2.13
V(N1,S8)	**g**		1.94	1.93
V(N1,B9)	**h**			2.35

**Table 2:** B3LYP/6-31G(d) electronic chemical potential μ, chemical hardness η, electrophilicity ω, and nucleophilicity *N*, in eV, of aziridine **22**, 2PA **20**, 2PTA **4**, 2PTA:BF_3_ **21**, and ketone **8**.

	μ	η	ω	*N*
2PTA:BF_3_ **21**	−4.53	5.91	1.73	1.64
2PTA **4**	−4.02	6.08	1.33	2.06
ketone **8**	−3.47	6.34	0.95	2.48
2PA **20**	−3.06	6.30	0.74	2.91
aziridine **22**	−2.22	8.82	0.28	2.49

**Table 3:** B3LYP/6-31G(d) nucleofugality Λ indices, in eV, of methylamine **29**, sulfonamides **25** and **28**, and the corresponding LA complexes **24**, **25**, and **26**.

Methyl Derivative	LG	Λ
Me-NHTos:BF_3_ **23**	NHTos:BF_3_	1.79
Me-NHMes:BF_3_ **24**	NHMes:BF_3_	1.32
Me-NHTos **25**	NHTos	1.29
Me-NHMes:BH_3_ **26**	NHMes:BH_3_	1.08
Me-Br **27**	Br	0.90
Me-NHMes **28**	NHMes	0.48
Me-NH_2_ **29**	NH_2_	0.23

**Table 4:** *ω*B97X-D/6-311G(d,p) relative enthalpies, ΔH, and Gibbs free energies, ΔG, in kcal·mol^−1^, and entropies, ΔS, in cal·mol^−1^K^−1^, calculated in DCM at 25 °C, of the stationary points involved in the two regioisomeric reaction paths associated with LA BF_3_ promoted formal 32CA reaction of 2PTA **4** with ketone **8**.

	ΔH	ΔS	ΔG
**MC**	−5.85	−34.38	4.40
**TS21-2**	6.42	−38.85	18.00
**TS21-3**	14.60	−40.52	26.68
**IN21-2**	−5.14	−54.82	11.21
**TSrot-2**	−3.71	−46.40	10.12
**IN21-3**	−2.94	−44.76	10.41
**TSrot-3**	1.65	−50.95	16.84
**IN22-2**	−12.13	−43.76	0.92
**IN22-3**	−4.24	−57.11	12.79
**TS22-2**	−7.36	−55.47	9.18
**TS22-3**	−2.56	−66.21	17.18
**IN23-2**	−15.45	−66.25	4.30
**IN23-3**	−8.86	−67.96	11.41
**9**	−19.73	−54.08	−3.60

**Table 5:** M06-2X/6-311G(d,p) gas-phase ξ $E_{intra}^{X}$ intra-atomic, ξ$E_{inter}^{X}$ inter-atomic, and ξ$E_{total}^{X}$ total energies, in kcal·mol^−1^, of the three frameworks *f*(X) in which the TSs have been divided relative to their GSs, where X represents the **A** ketone, **B** phenyl ethylene, and **C** sulfonamide frameworks. The sum of the ξ$E_{total}^{X}$ energies of the three frameworks, denoted as ξ$E_{total}^{A+B+C}$, represents the RIAE activation energies.

	LA	ƒ(X)	$\xi E_{intra}^{X}$	$\xi E_{inter}^{X}$	$\xi E_{total}^{X}$	$\xi E_{total}^{A+B+C}$
**TS3**		**A**	−15.67	43.37	27.69	41.04
	-	**B**	45.43	−28.43	17.00	
		**C**	203.30	−206.96	−3.66	
**TS4**		**A**	−15.20	39.04	23.84	25.47
	BH_3_	**B**	35.70	−22.07	13.63	
		**C**	298.67	−310.68	−12.01	
**TS5**		**A**	−12.01	29.85	17.84	18.23
	BF_3_	**B**	30.30	−10.83	19.47	
		**C**	274.59	−293.67	−19.08	

## Data Availability

The data underlying this study are available in the published article and its Supplementary Material.

## Supplementary Materials

The following supporting information can be downloaded at: https://www.mdpi.com/article/10.3390/molecules31030509/s1. Theoretical background of the Relative Interacting Atomic Energy (RIAE) Analysis [24,28,29,40,60,62,63,64,65,66,67]; Figure S1: Figure with the *ω*B97X-D/6-311G(d,p) optimized geometries of the *cis* and *trans* conformations of (2*R*)-2PTA **4**; Figure S2: Figure with the *ω*B97X-D/6-311G(d,p) optimized geometries in DCM of **MC**; Figure S3: Figure with the *ω*B97X-D/6-311G(d,p) optimized geometries in DCM of **TS21-2’** and **TS21-3**’; Table S1: Table with the *ω*B97X-D/6-311G(d,p) enthalpies, entropies, and Gibbs free energies of the stationary points involved in the non-catalyzed formal 32CA reaction of 2PTA **4** with ketone **8**; Table S2: Table with the *ω*B97X-D/6-311G(d,p) enthalpies, entropies, and Gibbs free energies of the stationary points involved in the BF_3_ LA-promoted formal 32CA reaction of 2PTA:BF_3_ complex **21** with ketone **8**; Table S3: Table with the *ω*B97X-D/6-311G(d,p) enthalpies, entropies, and Gibbs free energies of the reagents and product involved in the formal 32CA reaction of 2PTA **4** with ketone **8**, yielding 1,3-oxazolidine **9**; Table S4: Table with the M06-2X/6-311G(d,p) gas phase total and relative energies of the reagents and TSs involved in the LA-catalyzed aziridine ring-opening of 2PMA **19** in the presence of ketone **8**, as well as in the absence of and in the presence of the BH_3_ and BF_3_ Las; Table S5: Table with the sum of the M06-2X/6-311G(d,p) gas-phase natural changes in the **A**, **B**, and **C** frameworks of the TSs associated with the LA-catalyzed aziridine ring-opening of 2PMA **19** in the presence of ketone **8**, as well as in the absence of and in the presence of the BH_3_ and BF_3_ Las; *ω*B97X-D/6-311G(d,p) computed total energies in DCM, single imaginary frequency, and Cartesian coordinates of the stationary points involved in the non-catalyzed opening of the aziridine ring of 2PTA **4** in presence of ketone **8**. *ω*B97X-D/6-311G(d,p) computed total energies in DCM, single imaginary frequency, and Cartesian coordinates of the stationary points involved in the LA-promoted formal 32CA reaction of the 2PTA:BF_3_ complex **21** by ketone **8**. M06-2X/6-311G(d,p)-computed total energies in DCM, single imaginary frequency, and Cartesian coordinates of the reagents and TSs involved in the aziridine ring-opening of 2PMA **19** in the presence of ketone **8**, as well as in the absence of and in the presence of the BH_3_ and BF_3_ LAs.
