# Supplementary material for: Mechanistic Insights into Lewis Acid-Catalyzed Formal [3 + 2] Cycloadditions of Aziridines: A Molecular Electron Density Theory Study
Source: Molecules. 2026 Feb 2;31(3):509. doi: 10.3390/molecules31030509 (PMC12899876; doi:10.3390/molecules31030509)
Supplement: Supplementary file 1 [file molecules-31-00509-s001.zip › molecules-4089051-supplementary.pdf]

Mechanistic Insights into Lewis Acid-Catalyzed Formal [3 + 2] Cycloadditions of Aziridines: A Molecular Electron Density Theory Study

Luis R. Domingo [1,*], Patricia Pérez [2,*] and Maria José Aurell [3]

[1] Independent Researcher, Av. Tirso de Molina 20, 46015 Valencia, Spain
[2] Facultad de Ciencias, Escuela de Química y Farmacia, Universidad San Sebastián, Campus Ciudad Universitaria, Av. del Cóndor 720, Ciudad Empresarial, Huechuraba, Santiago 8580704, Chile
[3] Department of Organic Chemistry, University of Valencia, Dr. Moliner 50, 46100 Burjassot, Spain; maria.j.aurell@uv.es
* Correspondence: luisrdomingo@gmail.com (L.R.D.); patricia.perez@uss.cl (P.P.)

Index

S2 Theoretical background of the Relative Interacting Atomic Energy (RIAE) Analysis.

S5 Figure with the ωB97X-D/6-311G(d,p) optimized geometries of the *cis* and *trans* conformations of (*2R*)-2PTA **4**.

S6 Figure with the ωB97X-D/6-311G(d,p) optimized geometries in DCM of **MC**.

S7 Figure with the ωB97X-D/6-311G(d,p) optimized geometries in DCM of **TS21-2'** and **TS21-3**'.

S8 Table with the ωB97X-D/6-311G(d,p) enthalpies, entropies, and Gibbs free energies of the stationary points involved in the non-catalyzed formal 32CA reaction of 2PTA **4** with ketone **8**.

S8 Table with the ωB97X-D/6-311G(d,p) enthalpies, entropies, and Gibbs free energies of the stationary points involved in the formal 32CA reaction of 2PTA:BF_3 complex **21** with ketone **8**.

S9 Table with the ωB97X-D/6-311G(d,p) enthalpies, entropies, and Gibbs free energies of the reagents and product involved in the formal 32CA reaction of 2PTA **4** with ketone **8** yielding 1,3-oxazolidine **9**.

S9 Table with the M06-2X/6-311G(d,p) gas phase total and relative energies of the reagents and TSs involved in the LA-catalyzed aziridine ring-opening of 2PMA **19** in presence of ketone **8**, in absence and in the presence of the BH_3 and BF_3 LAs.

S9 Table with the sum of the M06-2X/6-311G(d,p) gas-phase natural charges in the **A**, **B**, and **C** frameworks of the TSs associated with the LA-catalyzed aziridine ring-opening of 2PMA **19** in the presence of ketone **8**, in the absence and in the presence of the BH_3 and BF_3 LAs.

S10 ωB97X-D/6-311G(d,p) computed total energies in DCM, single imaginary frequency, and Cartesian coordinates of the stationary points involved in the non-catalyzed opening of the aziridine ring of 2PTA **4** in the presence of ketone **8**.

S15 ωB97X-D/6-311G(d,p) computed total energies in DCM, single imaginary frequency, and Cartesian coordinates of the stationary points involved in the LA-promoted formal 32CA reaction of 2PTA:BF_3 complex **21** by ketone **8**.

S32 M06-2X/6-311G(d,p) computed total energies in DCM, single imaginary frequency, and Cartesian coordinates of the reagents and TSs involved in the aziridine ring-opening of 2PMA **19** in the presence of ketone **8**, in the absence and in the presence of the BH_3 and BF_3 LAs.

1. Theoretical background of the Relative Interacting Atomic Energy (RIAE) Analysis

The Interacting Quantum Atoms [1] (IQA), based on AIM [2,3], divides the E_{total}^{IQA} total energy into two main energy contributions: the E_{intra}^{A} intra-atomic energies and the E_{inter}^{AB} interatomic energies (see Equation S1). The E_{inter}^{AB} energies are, in turn, divided into four additional electrostatic terms: the V_{ne}^{AB} and V_{en}^{AB} nuclei-electron interactions, the V_{ee}^{AB} electron-electron interactions, and the V_{nn}^{AB} nuclei-nuclei interactions (see Equation S3).

$$E_{total}^{IQA} = \sum E_{intra}^{A} + \sum E_{inter}^{AB} \quad \text{(S1)}$$

$$E_{intra}^{A} = T(A) + V_{ne}^{A} + V_{ee}^{A} \quad \text{(S2)}$$

$$E_{inter}^{AB} = ½V_{ne}^{AB} + ½V_{en}^{AB} + ½V_{ee}^{AB} + V_{nn}^{AB} \quad \text{(S3)}$$

Thanks to the additivity of the topological atoms [4], an interacting quantum fragment (IQF) approach has been recently introduced [5], which allows the grouping of the IQA energy into convenient fragments of the system. This enables a more chemically meaningful analysis of the interactions that occur between the atoms forming groups. In this sense, for Relative Interacting Atomic Energy [6] (RIAE) analysis the atoms belonging to the reagents and the TSs of bimolecular of organic reactions such as Diels-Alder reactions [7], [3+2] cycloaddition reactions [8], or Alder-ene reactions [9] were regrouped into the two interacting frameworks f(X) **A** and **B**. Recently, the atoms involved in the reagents and the TSs participating in SN reactions have been regrouped into ~~the~~ three frameworks f(X), **A**, **B** and **C**, in which the TSs are topologically shared, i.e., ketone **A**, phenylethylene **B**, and sulfonamide **C** [10,11].

By default, the sum of all IQA atomic energies belonging to the considered framework f(X) (where X represents either the **A**, **B**, and **C** frameworks) at the TSs, and those of the corresponding reagents at the ground states, is computed. The RIAEs, i.e., the relative ξE_{total}^{X} total, ξE_{intra}^{X} intra-atomic, and ξE_{inter}^{X} interatomic energies, are obtained using Equations S4-S6. The symbol ξ denotes the IQA energy differences between the TS and the ground states of the three-interacting frameworks f(X); i.e. f(ketone), f(phenylethylene) and f(sulfonamide) in the SN reactions.

$$\xi E_{total}^{X} = \xi E_{intra}^{X} + \xi E_{inter}^{X} \quad \text{(S4)}$$

$$\xi E_{intra}^{X} = \sum E_{intra}^{X(TS)} - \sum E_{intra}^{X(MC)} \quad \text{(S5)}$$

$$\xi E_{inter}^{X} = \sum E_{inter}^{X(TS)} - \sum E_{inter}^{X(MC)} \quad \text{(S6)}$$

The RIAE analysis provides a measure of how much the three interacting frameworks f(X) are destabilized (resulting in positive relative energies) or stabilized (resulting in negative relative energies) when going from their reagents to the TSs. The sum of the ξE_{total}^{X} energies of the three interacting frameworks, ξE_{total}^{A+B+C}, provides the RIAE activation energy of the SN reactions obtained through the present EDA [10,11].

References

1. Blanco, M. A.; Martín Pendás, A.; Francisco, E. Interacting Quantum Atoms: A Correlated Energy Decomposition Scheme Based on the Quantum Theory of Atoms in Molecules, *J. Chem. Theory Comput.* **2005**, *1*, 1096–1109.
2. Bader, R.F.W.; Tang, Y.H.; Tal, Y.; Biegler-König, F.W. Properties of atoms and bonds in hydrocarbon molecules, *J. Am. Chem. Soc*. **1982**, *104*, 946–952.
3. Bader, R.F.W. In *Atoms in Molecules: A Quantum Theory*, Oxford University Press, Oxford, New York, 1994.
4. Martín Pendás, A.; Blanco, M.A.; Francisco, E. Chemical Fragments in Realpace: Definitions, Properties and Energetic Decompositions, *J. Comput. Chem.* **2007**, *28*, 161–184.
5. Triestram, L.; Falcioni, F.; Popelier P.L.A., Interacting Quantum Atoms and Multipolar Electrostatic Study of XH···π Interactions, *ACS Omega* **2023,** *8,* 34844–34851.
6. Domingo, L. R.; Ríos-Gutiérrez, M.; Pérez, P. Understanding the Electronic Effects of Lewis Acid Catalysts in Accelerating Polar Diels–Alder Reactions, *J. Org. Chem.* **2024**, *89*, 12349−12359.
7. Domingo, L.R. Performing a Relative Interacting Atomic Energy Analysis of the Diels-Alder Reaction between Butadiene and Ethylene. *Sci. Rad.* **2025**, *4*, 287-306.
8. Domingo, L.R.; Pérez, P. How Different Are Nitrile Oxides from Nitrones in *zw-type* [3+2] Cycloaddition Reactions? A Molecular Electron Density Theory Study. *J. Org. Chem.* **2025**, *90*, 3936−3950.
9. Domingo, L.R.; Pérez, P. Intramolecular versus Intermolecular Diels-Alder Reactions: Insights from Molecular Electron Density Theory. *Molecules* **2025**, *30*, 4289.
10. Domingo, L.R.; Pérez, P.; Ríos-Gutiérrez, M.; José Aurell, M. Advanced Molecular Electron Density Theory Study of the Substituent Effects in Nucleophilic Substitution Reactions. *ACS Omega* **2025***, 10,* 30194–30206.
11. Domingo, L.R.; Aurell, M. J.; Pérez, P. Unveiling the Electronic Effects of the Lewis Acids in Nucleophilic Substitution Reactions: A Molecular Electron Density Theory Study. *J. Org. Chem.,* **2026**, https://doi.org/10.1021/acs.joc.5c02214.

.

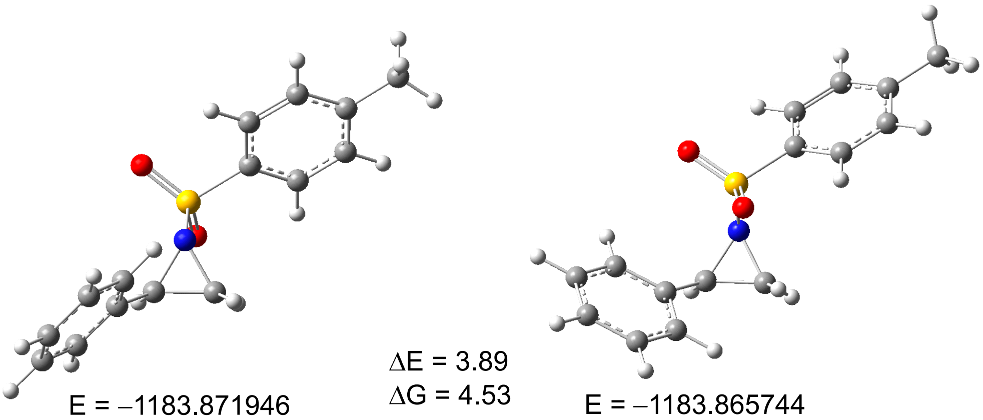

ΔE = 3.89
ΔG = 4.53

E = –1183.871946
G = –1183.648425
trans (2*R*)-2PTA **4**

E = –1183.865744
G = –1183.641210
cis (2*R*)-2PTA **4**

Figure S1. *ω*B97X-D/6-311G(d,p) optimized geometries of the *cis* and *trans* conformations of (*2R*)-2PTA **4**. Total electronic energies E and Gibbs free energies G, computed in DCM at 25 ºC, are given in a.u., and relative energies ΔE and Gibbs free energies ΔG are given in kcal·mol^{-1}.

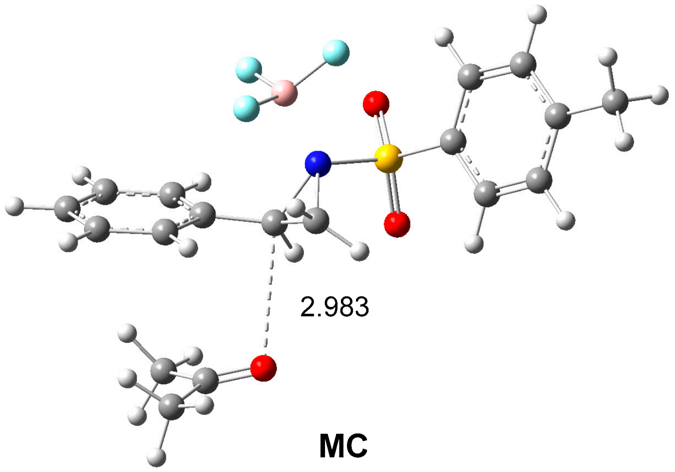

MC

Figure S2. *ω*B97X-D/6-311G(d,p) optimized geometries in DCM of **MC**. The distance is given in Angstroms.

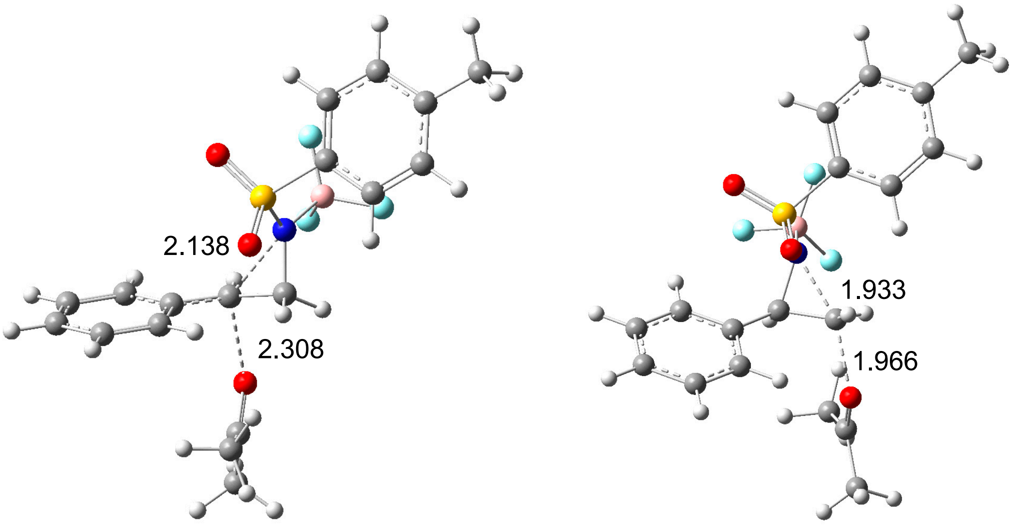

TS21-2' **TS21-3'**

Figure S3. *ω*B97X-D/6-311G(d,p) optimized geometries in DCM of **TS21-2’** and **TS21-3’**. The distances are given in Angstroms.

Table S1. ωB97X-D/6-311G(d,p) enthalpies, H, and Gibbs free energies, G, in a.u., as well as entropies, S, in cal·mol^{-1}·K^{-1}, computed in DCM at 25 ºC, of the stationary points involved in the non-catalyzed formal 32CA reaction of 2PTA **4** with ketone **8**.

	H	S	G
trans 2PTA **4**	-1183.582475	138.77	-1183.648411
cis 2PTA **4**	-1183.576184	136.86	-1183.641210
ketone **8**	-193.060027	71.77	-193.094127
**TS1-2**	-1376.598842	167.88	-1376.674118
**TS1-3**	-1376.594354	161.23	-1376.675445
**IN1-2**	-1376.604214	160.05	-1376.680258
**IN1-3**	-1376.604429	167.39	-1376.683959

Table S2. ωB97X-D/6-311G(d,p) enthalpies, H, and Gibbs free energies, G, in a.u., as well as entropies, S, in cal·mol^{-1}·K^{-1}, computed in DCM at 25 ºC, of the stationary points involved in the formal 32CA reaction of 2PTA:BF_3 complex **21** with ketone **8**.

	H	S	G
2PTA:BF_3 **21**	-1508.151384	157.27	-1508.226110
ketone **8**	-193.060027	71.77	-193.094127
**MC**	-1701.220729	194.66	-1701.313220
**TS21-2**	-1701.201182	190.19	-1701.291547
**TS21-3**	-1701.188138	188.52	-1701.277712
**TS21-2'**	-1701.199587	190.25	-1701.289981
**TS21-3'**	-1701.187958	184.37	-1701.275557
**IN21-2**	-1701.219603	174.22	-1701.302378
**TSrot-2**	-1701.217327	182.64	-1701.304105
**IN21-3**	-1701.216099	184.28	-1701.303655
**TSrot-3**	-1701.208780	178.09	-1701.293394
**IN22-2**	-1701.230742	185.28	-1701.318775
**IN22-3**	-1701.218162	171.93	-1701.299851
**TS22-2**	-1701.223145	173.57	-1701.305614
**TS22-3**	-1701.215487	162.83	-1701.292855
**IN23-2**	-1701.236039	162.79	-1701.313384
**IN23-3**	-1701.225523	161.08	-1701.302055

Table S3. ωB97X-D/6-311G(d,p) enthalpies, H, and Gibbs free energies, G, in a.u., as well as entropies, S, in cal·mol^{-1}·K^{-1}, computed in DCM at 25 ºC, of the reagents and product involved in the formal 32CA reaction of 2PTA **4** with ketone **8** yielding 1,3-oxazolidine **9**.

	H	S	H
2PTA **4**	-1183.582475	138.80	-1183.648425
ketone **8**	-193.060026	71.78	-193.094129
1,3-oxazolidine **9**	-1376.673938	156.50	-1376.748295

Table S4. M06-2X/6-311G(d,p) gas phase total (E, in a.u.) and relative (ΔE in kcal·mol^{-1}) energies of the reagents and TSs involved in the LA-catalyzed aziridine ring-opening of 2PMA **19** in presence of ketone **8**, in the absence and in presence of the BH_3 and BF_3 LAs.

		E	ΔE
ketone **8**		-193.115149	
2PMA	**19**	-952.776987	
**TS3**		-1145.826991	40.88
2PMA:BH_3	**19**:BH_3	-979.413699	
**TS4**		-1172.488220	25.49
2PMA:BF_3	**19**:BF_3	-1277.361335	
**TS5**		-1470.447198	18.38

Table S5. Sum of the M06-2X/6-311G(d,p) gas-phase natural changes in the **A**, **B**, and **C** frameworks as an average of the number of electrons of the TSs associated with the LA-catalyzed aziridine ring-opening of 2PMA **19** in presence of ketone **8**, in absence and in the presence of the BH_3 and BF_3 LAs.

fragments	**TS3**	**TS4**	**TS5**
**A**	0.34	0.28	0.23
**B**	0.56	0.71	0.78
**C**	-0.90	-0.99	-1.01

ωB97X-D/6-311G(d,p) computed total energies in DCM, single imaginary frequency, and Cartesian coordinates of the stationary points involved in the non-catalyzed opening of the aziridine ring of 2PTA **4** in presence of ketone **8**.

2PTA **4**
E(RwB97XD) = -1183.87194624 A.U.

6	1.813723	-0.761009	-0.577447
6	0.783469	-0.331144	-1.561777
7	0.538592	-0.179894	-0.125965
1	1.907066	-1.838379	-0.469860
1	0.247826	-1.116105	-2.085537
1	0.901281	0.610961	-2.083961
16	-0.559266	-1.231729	0.599473
8	-0.587792	-2.515141	-0.090173
8	-0.256961	-1.206041	2.018443
6	-2.077899	-0.377980	0.284795
6	-2.387144	0.750410	1.037664
6	-3.564225	1.428719	0.770531
6	-4.434358	0.997075	-0.235476
6	-4.095398	-0.136777	-0.973565
6	-2.919017	-0.829938	-0.722507
1	-1.720186	1.086741	1.821868
1	-3.814176	2.309115	1.352225
1	-4.759149	-0.484794	-1.757125
1	-2.661602	-1.713665	-1.292478
6	3.052269	0.019351	-0.305202
6	4.286490	-0.623514	-0.374233
6	3.005663	1.381425	-0.011520
6	5.461813	0.087529	-0.161575
1	4.328735	-1.685402	-0.593498
6	4.180256	2.090186	0.203178
1	2.044685	1.878013	0.062497
6	5.411339	1.446279	0.126601
1	6.416720	-0.422458	-0.216535
1	4.134655	3.148072	0.435482
1	6.326969	2.001002	0.296425
6	-5.722911	1.728900	-0.492176
1	-6.104073	1.523976	-1.493684
1	-6.485287	1.414999	0.227165
1	-5.591741	2.807223	-0.382709

ketone **8**
E(RwB97XD) = -193.150150153 A.U.

8	0.000018	1.395282	-0.000006
6	0.000000	0.184746	-0.000013
6	1.282542	-0.612084	0.002251
1	1.376484	-1.147075	-0.947745
1	2.137475	0.049895	0.132727
1	1.264267	-1.365800	0.793771
6	-1.282552	-0.612065	-0.002283
1	-1.264140	-1.366374	-0.793222
1	-1.376725	-1.146336	0.948105
1	-2.137449	0.049850	-0.133319

TS1-2
E(RwB97XD) = -1376.97871433 A.U.
Imaginary frequency -329.9204 cm-1

6	1.437475	0.092195	-0.435863
6	0.234174	0.246020	0.418855
7	-0.220157	-1.134690	0.335076
1	1.194672	-0.011815	-1.486531
1	-0.442085	1.005144	0.009741
1	0.460550	0.509345	1.454078
16	-1.302807	-1.399280	-0.802703
8	-0.970593	-0.786129	-2.103171
8	-1.593298	-2.834044	-0.822865
6	-2.803293	-0.565551	-0.266757
6	-3.388278	-0.938076	0.941515
6	-4.530342	-0.287072	1.376279
6	-5.104843	0.744340	0.624913
6	-4.498990	1.105431	-0.575524
6	-3.352041	0.457066	-1.024922
1	-2.947864	-1.730162	1.535840
1	-4.986511	-0.579662	2.316566
1	-4.925891	1.905100	-1.171871
1	-2.883563	0.735769	-1.960564
6	2.660202	-0.583079	0.016141
6	2.807276	-1.057397	1.322439
6	3.714745	-0.726580	-0.889891
6	3.997084	-1.648484	1.716728
1	1.982327	-0.991984	2.020736
6	4.911268	-1.301499	-0.488074
1	3.595715	-0.371993	-1.907819
6	5.053398	-1.761115	0.817019
1	4.101975	-2.025043	2.727098
1	5.727628	-1.400851	-1.193257
1	5.984289	-2.218828	1.131114
8	1.998465	1.873964	-0.817330
6	2.641118	2.653476	-0.112946
6	3.114290	2.320179	1.264149
1	4.009785	1.696410	1.169212
1	3.378820	3.222393	1.812726
1	2.369816	1.747756	1.814409
6	2.985797	3.992605	-0.670341
1	2.472864	4.755934	-0.077109
1	4.058559	4.167775	-0.552834
1	2.692959	4.069504	-1.714853
6	-6.357852	1.427658	1.104109
1	-6.551784	2.343082	0.542611
1	-7.223965	0.770012	0.984118
1	-6.286006	1.682393	2.164252

TS1-3
E(RwB97XD) = -1376.97532236 A.U.
Imaginary frequency -512.8093 cm-1

6	1.307106	0.117803	0.166295

6	1.185800	-0.956779	-0.838937
7	-0.110467	0.409297	-0.070132
1	1.528197	-0.269452	1.166197
1	0.597850	-1.814580	-0.547308
1	1.132222	-0.647103	-1.873700
16	-1.053874	-0.187500	1.087181
8	-0.781335	-1.616910	1.362002
8	-1.074910	0.651784	2.293988
6	-2.661044	-0.072746	0.323942
6	-3.639436	0.719677	0.900824
6	-4.899797	0.779564	0.314229
6	-5.190380	0.057622	-0.840225
6	-4.183999	-0.736418	-1.401835
6	-2.926353	-0.807195	-0.828478
1	-3.413893	1.282755	1.797794
1	-5.666042	1.401253	0.764749
1	-4.393138	-1.305590	-2.301696
1	-2.154272	-1.423168	-1.273519
6	2.198082	1.300668	-0.121239
6	3.238146	1.259936	-1.044173
6	1.967903	2.477195	0.592934
6	4.039845	2.380643	-1.250106
1	3.434440	0.355673	-1.608444
6	2.767761	3.591753	0.391058
1	1.145227	2.510540	1.299491
6	3.808882	3.546578	-0.533688
1	4.844771	2.337935	-1.975278
1	2.577752	4.500397	0.951230
1	4.432393	4.418605	-0.695171
8	2.759795	-1.873880	-1.066252
6	3.238884	-2.609583	-0.198034
6	4.533615	-3.288526	-0.488337
1	4.992943	-2.885014	-1.387918
1	5.203170	-3.194895	0.369441
1	4.333766	-4.356314	-0.623221
6	2.609618	-2.843480	1.134486
1	2.807546	-3.863662	1.464504
1	3.114872	-2.176923	1.843428
1	1.541286	-2.631999	1.163665
6	-6.551656	0.119388	-1.479476
1	-6.479957	0.473165	-2.511480
1	-7.012885	-0.871657	-1.505525
1	-7.217259	0.790409	-0.934551

IN1-2

E(RwB97XD) = -1376.98623291 A.U.

6	1.470815	0.298203	-0.434360
6	0.306262	0.205003	0.557708
7	-0.174139	-1.154867	0.650477
1	1.105261	-0.031422	-1.404715
1	-0.451028	0.935043	0.229887
1	0.629332	0.522017	1.554444
16	-1.203459	-1.564839	-0.463016
8	-0.820801	-1.169278	-1.838562

8	-1.540592	-2.984102	-0.296330
6	-2.725095	-0.642158	-0.152925
6	-3.198840	-0.538510	1.152123
6	-4.380247	0.141427	1.403119
6	-5.108756	0.729353	0.364404
6	-4.613312	0.622315	-0.933341
6	-3.428696	-0.058695	-1.196259
1	-2.630398	-0.977077	1.964316
1	-4.744392	0.222124	2.422538
1	-5.158146	1.079203	-1.753244
1	-3.041838	-0.133376	-2.205041
6	2.775835	-0.370898	-0.096962
6	2.937896	-1.119892	1.066048
6	3.846255	-0.244834	-0.984420
6	4.163669	-1.710256	1.349357
1	2.099254	-1.264918	1.734857
6	5.073872	-0.823272	-0.693221
1	3.720782	0.314653	-1.905971
6	5.235382	-1.555418	0.478515
1	4.279100	-2.295113	2.254365
1	5.900255	-0.711887	-1.385330
1	6.191118	-2.013536	0.705310
8	1.702243	1.771629	-0.746282
6	2.303654	2.616379	-0.038647
6	2.981436	2.326100	1.243227
1	3.981806	1.948981	0.995123
1	3.095438	3.245158	1.814758
1	2.481119	1.561511	1.830334
6	2.338232	3.988972	-0.582182
1	1.738119	4.622535	0.080009
1	3.361751	4.369134	-0.533682
1	1.947619	4.028662	-1.595526
6	-6.401190	1.447432	0.650292
1	-6.733171	2.027789	-0.212326
1	-7.191881	0.733065	0.898508
1	-6.295627	2.125445	1.500832

IN1-3
E(RwB97XD) = -1376.98746938 A.U.

6	1.319746	0.052323	-0.008827
6	1.569210	-0.961515	-1.124226
7	-0.095247	0.377258	-0.098712
1	1.586184	-0.430495	0.945124
1	0.839875	-1.764435	-1.065202
1	1.556226	-0.500153	-2.107976
16	-0.985101	-0.490934	0.865583
8	-0.757816	-1.959476	0.720768
8	-0.964290	-0.081093	2.286439
6	-2.628256	-0.147159	0.252284
6	-3.626539	0.219889	1.139956
6	-4.918703	0.431098	0.667848
6	-5.224784	0.278584	-0.681748
6	-4.199559	-0.092971	-1.558539
6	-2.910463	-0.308112	-1.101322

1	-3.388723	0.344826	2.189034
1	-5.697980	0.722506	1.364251
1	-4.418342	-0.212520	-2.614880
1	-2.119031	-0.583428	-1.787614
6	2.159755	1.317490	-0.085198
6	3.202510	1.504744	-0.987529
6	1.871655	2.335697	0.826571
6	3.942105	2.686482	-0.982586
1	3.459291	0.737527	-1.708874
6	2.609835	3.508759	0.838120
1	1.050089	2.194134	1.520347
6	3.650292	3.690152	-0.071031
1	4.746555	2.817326	-1.697818
1	2.371936	4.288007	1.553698
1	4.225036	4.609398	-0.067371
8	2.921358	-1.574926	-1.057660
6	3.235274	-2.478848	-0.239104
6	4.620365	-2.977917	-0.354415
1	5.240048	-2.296395	-0.932235
1	5.038973	-3.161634	0.635643
1	4.570695	-3.945813	-0.867088
6	2.328875	-3.037946	0.780307
1	2.488508	-4.116515	0.836057
1	2.669496	-2.632626	1.742110
1	1.272701	-2.791318	0.658579
6	-6.620193	0.506915	-1.199047
1	-6.629446	1.292542	-1.959474
1	-7.015903	-0.400740	-1.663137
1	-7.298611	0.801907	-0.396991

ωB97X-D/6-311G(d,p) computed total energies in DCM, single imaginary frequency, and Cartesian coordinates of the stationary points involved in the LA-promoted formal 32CA reaction of 2PTA:BF_3 complex **21** by ketone **8**.

2PTA:BF_3 **21**
E(RwB97XD) = -1508.45948985 A.U.

6	-1.816573	1.130365	-0.509311
6	-0.983572	0.958522	-1.711880
1	-1.763069	2.125288	-0.075694
1	-0.462330	1.826114	-2.094776
1	-1.212921	0.166734	-2.412605
7	-0.484701	0.433288	-0.416171
16	0.683981	1.433650	0.488405
8	0.700177	2.704170	-0.205917
8	0.234702	1.348763	1.856328
6	2.222090	0.617022	0.284458
6	2.636052	-0.287850	1.253729
6	3.849458	-0.930889	1.077988
6	4.639282	-0.686343	-0.047359
6	4.189668	0.229762	-1.002537
6	2.982953	0.888515	-0.847367
1	2.016210	-0.489692	2.116736
1	4.185549	-1.640654	1.825019
1	4.793829	0.428763	-1.880293
1	2.642231	1.601985	-1.586605
5	-0.339988	-1.234538	-0.308151
6	-3.065950	0.390056	-0.183516
6	-3.979438	0.062915	-1.178487
6	-3.341623	0.081866	1.146436
6	-5.166111	-0.578926	-0.845721
1	-3.763423	0.303429	-2.213334
6	-4.522755	-0.568043	1.475495
1	-2.623610	0.341030	1.916886
6	-5.436862	-0.898142	0.479623
1	-5.876553	-0.834181	-1.623238
1	-4.730987	-0.816833	2.509463
1	-6.359920	-1.404329	0.737606
9	-1.440292	-1.756178	-0.950411
9	0.826356	-1.537508	-0.980051
9	-0.290858	-1.530309	1.034939
6	5.964959	-1.373402	-0.217002
1	6.188135	-1.545500	-1.271246
1	6.765259	-0.750634	0.194248
1	5.985392	-2.330322	0.306456

8
E(RwB97XD) = -193.150150153 A.U.

8	0.000018	1.395282	-0.000006
6	0.000000	0.184746	-0.000013
6	1.282542	-0.612084	0.002251
1	1.376484	-1.147075	-0.947745
1	2.137475	0.049895	0.132727
1	1.264267	-1.365800	0.793771

6	-1.282552	-0.612065	-0.002283
1	-1.264140	-1.366374	-0.793222
1	-1.376725	-1.146336	0.948105
1	-2.137449	0.049850	-0.133319

MC

E(RwB97XD) = -1701.62128706 A.U.

6	-1.082859	0.255819	0.212211
6	-0.116541	0.713670	-0.791587
7	0.257130	-0.439810	0.072088
1	-1.112760	0.870288	1.104430
1	0.406434	1.640442	-0.595986
1	-0.225778	0.430581	-1.828963
16	1.413970	-0.090503	1.380544
8	0.926756	1.124999	1.997905
8	1.517871	-1.314804	2.131754
6	2.921207	0.270875	0.539006
6	3.840407	-0.747333	0.310299
6	5.036979	-0.431130	-0.309296
6	5.326072	0.878510	-0.701923
6	4.385992	1.879296	-0.448737
6	3.184875	1.588883	0.177627
1	3.617924	-1.762811	0.605012
1	5.760340	-1.217276	-0.493464
1	4.598546	2.902345	-0.736865
1	2.478245	2.377582	0.402318
5	0.329761	-1.965396	-0.616352
6	-2.339613	-0.488257	-0.070962
6	-2.998229	-0.347241	-1.287477
6	-2.910242	-1.258836	0.939380
6	-4.222540	-0.971423	-1.493504
1	-2.564401	0.259948	-2.073234
6	-4.126773	-1.891651	0.730170
1	-2.397025	-1.363440	1.888511
6	-4.786990	-1.745341	-0.486271
1	-4.733919	-0.853207	-2.441629
1	-4.563614	-2.494604	1.517337
1	-5.740869	-2.233919	-0.647828
9	-0.324138	-1.853833	-1.828916
9	1.672539	-2.241946	-0.785167
9	-0.289581	-2.825309	0.255680
8	-2.538397	2.858303	0.280279
6	-3.704107	2.607749	0.057365
6	-4.583612	1.924778	1.072327
1	-4.891100	0.949813	0.681151
1	-4.046790	1.787771	2.009893
1	-5.492804	2.508507	1.239818
6	-4.352809	2.964281	-1.256885
1	-4.885724	2.101112	-1.663593
1	-5.093253	3.752740	-1.090640
1	-3.603222	3.313830	-1.965524
6	6.641624	1.206432	-1.350408
1	7.405483	1.372533	-0.584752
1	6.983217	0.386711	-1.984767
1	6.572558	2.112082	-1.954664

TS21-2
E(RwB97XD) = -1701.60072812 A.U.
Imaginary frequency -86.5101 cm-1

6	-1.471002	0.263715	0.435609
6	-0.276352	0.363940	-0.424600
7	0.386501	-0.811504	0.147223
1	-1.314475	0.621699	1.446191
1	0.266686	1.292595	-0.256479
1	-0.448864	0.221460	-1.485978
16	1.462741	-0.507716	1.372804
8	0.916048	0.577992	2.178285
8	1.778839	-1.769312	2.007881
6	2.915482	0.132614	0.568020
6	3.785270	-0.743433	-0.075482
6	4.900846	-0.229049	-0.713262
6	5.161748	1.145118	-0.724554
6	4.271718	1.997978	-0.074753
6	3.147590	1.501591	0.573965
1	3.578293	-1.804910	-0.082806
1	5.583629	-0.906115	-1.215262
1	4.457901	3.066310	-0.070053
1	2.468400	2.166096	1.092877
5	0.314443	-2.214013	-0.604576
6	-2.675125	-0.435585	0.165671
6	-2.957090	-1.018020	-1.085277
6	-3.649958	-0.462557	1.182771
6	-4.191835	-1.591717	-1.310212
1	-2.199911	-1.041047	-1.856841
6	-4.888143	-1.027056	0.944814
1	-3.424233	-0.020436	2.146307
6	-5.156363	-1.587712	-0.302354
1	-4.411732	-2.046219	-2.267808
1	-5.641767	-1.039088	1.721778
1	-6.125941	-2.034551	-0.489675
9	-0.146546	-1.942560	-1.908798
9	1.583912	-2.787971	-0.666528
9	-0.582154	-3.054195	0.050358
8	-2.065478	2.544670	0.229848
6	-3.037642	3.070816	-0.282954
6	-3.913903	2.339942	-1.264658
1	-4.614281	1.714745	-0.699735
1	-4.494597	3.029296	-1.877113
1	-3.314710	1.686368	-1.897446
6	-3.415778	4.481538	0.060598
1	-3.303016	5.106375	-0.830632
1	-4.470972	4.521655	0.345168
1	-2.788979	4.864016	0.863976
6	6.390764	1.678200	-1.408930
1	6.347271	2.762728	-1.518666
1	7.286103	1.432714	-0.830348
1	6.510092	1.234144	-2.399865

TS21-3
E(RwB97XD) = -1701.58771963 A.U.

Imaginary frequency -521.9698 cm-1

6	-1.182947	0.130317	0.396463
6	-0.536386	1.065202	-0.533691
7	0.017889	-0.691982	0.107207
1	-1.135253	0.474536	1.425511
1	0.255859	1.692051	-0.150470
1	-0.535299	0.823220	-1.585076
16	1.186588	-0.750075	1.331920
8	0.785363	0.231989	2.332097
8	1.379004	-2.127994	1.732783
6	2.681572	-0.152681	0.579640
6	3.566415	-1.044340	-0.012108
6	4.739044	-0.554532	-0.565875
6	5.039335	0.808717	-0.538984
6	4.138149	1.678801	0.077092
6	2.962532	1.207651	0.641178
1	3.334874	-2.099316	-0.043293
1	5.435692	-1.246749	-1.025696
1	4.363934	2.738151	0.128376
1	2.289259	1.885687	1.151028
5	-0.042254	-1.861248	-0.993899
6	-2.531733	-0.468578	0.118126
6	-3.256601	-0.183776	-1.033683
6	-3.079268	-1.312534	1.082975
6	-4.518607	-0.737401	-1.218245
1	-2.844878	0.468411	-1.793267
6	-4.333789	-1.872592	0.895245
1	-2.511692	-1.538937	1.979517
6	-5.057721	-1.583296	-0.257629
1	-5.077483	-0.509878	-2.118524
1	-4.748147	-2.533451	1.647570
1	-6.039958	-2.017285	-0.405355
9	-0.636742	-1.308647	-2.136864
9	1.266008	-2.252944	-1.269371
9	-0.792523	-2.924045	-0.512964
8	-1.688840	2.594175	-0.926647
6	-2.310741	3.266115	-0.106252
6	-3.243134	4.323962	-0.591676
1	-3.326857	4.302223	-1.675857
1	-4.221953	4.183867	-0.124898
1	-2.870398	5.298235	-0.261184
6	-2.193189	3.048440	1.369034
1	-2.510287	3.929324	1.924706
1	-2.865181	2.224262	1.635525
1	-1.177705	2.772556	1.653150
6	6.298690	1.329665	-1.174908
1	6.635971	2.248852	-0.692872
1	7.102343	0.593125	-1.122146
1	6.123155	1.552955	-2.231713

TS21-2'

E(RwB97XD) = -1701.59896851 A.U.

Imaginary frequency -94.0288 cm-1

6	1.802692	-0.363771	0.502798
6	0.720816	-0.927661	-0.332521
7	-0.334674	-0.308368	0.467797
8	3.414245	-1.981604	0.168678
1	1.859078	-0.807215	1.487921
1	0.664888	-2.011887	-0.270109
1	0.710941	-0.599662	-1.368506
16	-1.087403	1.054996	-0.116905
8	-1.054806	2.097363	0.891007
8	-0.499658	1.335654	-1.423383
6	-2.775133	0.566104	-0.367164
6	-3.069167	-0.310477	-1.405695
6	-4.384573	-0.687387	-1.612094
6	-5.409775	-0.202457	-0.794167
6	-5.083079	0.676074	0.237974
6	-3.768377	1.061510	0.462500
1	-2.282552	-0.690214	-2.046059
1	-4.622848	-1.368689	-2.421607
1	-5.866441	1.063854	0.879751
1	-3.513084	1.735519	1.269337
5	-0.998175	-1.215864	1.582684
6	2.542976	0.832584	0.277078
6	2.551127	1.507934	-0.957875
6	3.339584	1.310556	1.334965
6	3.341712	2.628318	-1.122330
1	1.924262	1.167699	-1.770320
6	4.128613	2.431679	1.162839
1	3.328975	0.787014	2.283911
6	4.128048	3.087334	-0.066041
1	3.347160	3.152702	-2.069448
1	4.740114	2.798958	1.977096
1	4.744197	3.968550	-0.202746
9	-1.725985	-2.233006	0.954792
9	0.050249	-1.789061	2.325487
9	-1.815259	-0.432567	2.387927
6	4.187731	-2.128861	-0.761967
6	4.004146	-1.420685	-2.074643
1	4.548821	-0.471609	-2.025769
1	4.420766	-1.999192	-2.899644
1	2.953050	-1.205568	-2.260659
6	5.384904	-3.022529	-0.625360
1	5.253184	-3.889366	-1.280107
1	6.282411	-2.498959	-0.965242
1	5.501423	-3.353244	0.404870
6	-6.830771	-0.644329	-1.014342
1	-6.986836	-1.641970	-0.592979
1	-7.537532	0.035236	-0.535767
1	-7.066087	-0.697209	-2.079446

TS21-3'

E(RwB97XD) = -1701.58763045 A.U.
Imaginary frequency -512.5869 cm-1

6	-1.397225	0.144837	0.415556
6	-0.976199	1.254450	-0.444139
7	0.074394	0.003042	0.589576
8	-2.562065	2.175910	-1.152475
1	-1.782352	0.483258	1.374454
1	-0.605912	2.147527	0.033098
1	-0.665451	1.023447	-1.453295
16	0.914029	-1.155738	-0.302555
8	1.035550	-2.378480	0.468504
8	0.290163	-1.208028	-1.616104
6	2.523996	-0.434303	-0.483029
6	2.675198	0.706448	-1.261743
6	3.943586	1.233745	-1.437587
6	5.061537	0.631328	-0.853754
6	4.877342	-0.520174	-0.088522
6	3.612946	-1.056776	0.106104
1	1.820457	1.177759	-1.730687
1	4.069822	2.124328	-2.043324
1	5.734401	-1.004000	0.366556
1	3.468354	-1.942474	0.710614
5	0.527786	0.412573	2.066476
6	-2.229189	-1.007845	-0.071102
6	-2.886440	-0.995634	-1.296121
6	-2.377503	-2.098645	0.784327
6	-3.683003	-2.072822	-1.666743
1	-2.779893	-0.150635	-1.965254
6	-3.164389	-3.177459	0.407806
1	-1.861448	-2.098327	1.738518
6	-3.820044	-3.165529	-0.819356
1	-4.192239	-2.059230	-2.623419
1	-3.267833	-4.026694	1.073028
1	-4.437242	-4.006712	-1.113377
9	-0.131053	-0.429249	2.966944
9	1.899974	0.319623	2.196199
9	0.082390	1.737085	2.244500
6	-3.389465	2.757311	-0.453401
6	-4.574737	3.388221	-1.105250
1	-4.638209	3.105226	-2.153630
1	-5.484871	3.107213	-0.569947
1	-4.476547	4.474966	-1.019394
6	-3.284102	2.844594	1.036756
1	-3.755341	3.756433	1.402614
1	-3.846747	2.000012	1.451120
1	-2.257065	2.784124	1.393949
6	6.430115	1.230203	-1.029044
1	7.211662	0.480902	-0.893670
1	6.542723	1.676051	-2.019159
1	6.594059	2.020418	-0.290067

IN21-2

E(RwB97XD) = -1701.61995552 A.U.

6	-1.723268	0.847978	0.032732
6	-0.425541	0.434471	-0.656441
1	-1.498446	1.219036	1.030855
1	0.243132	1.300579	-0.706170
1	-0.646167	0.133649	-1.680387
7	0.175298	-0.706806	0.022893
16	1.116758	-0.332702	1.308412
8	0.549871	0.867815	1.926024
8	1.289205	-1.510351	2.137514
6	2.716216	0.133657	0.669414
6	3.579668	-0.854464	0.204642
6	4.809216	-0.483855	-0.311774
6	5.192429	0.859514	-0.379762
6	4.308268	1.827959	0.090472
6	3.069811	1.474148	0.614027
1	3.283033	-1.894795	0.241445
1	5.486927	-1.251043	-0.671041
1	4.590211	2.874766	0.054118
1	2.391561	2.227681	0.993834
5	0.499092	-1.899782	-0.948145
6	-2.797075	-0.200132	0.052853
6	-3.436515	-0.593823	-1.120780
6	-3.118699	-0.817449	1.257664
6	-4.404155	-1.586835	-1.083329
1	-3.175397	-0.130606	-2.065769
6	-4.077764	-1.822636	1.291267
1	-2.611292	-0.516913	2.167946
6	-4.724155	-2.203823	0.122091
1	-4.900442	-1.889804	-1.997609
1	-4.318669	-2.305800	2.230622
1	-5.474488	-2.985465	0.147673
9	-0.719381	-2.357058	-1.471531
9	1.293677	-1.415210	-2.012377
9	1.174877	-2.924154	-0.285976
8	-2.190392	2.017067	-0.774251
6	-3.010738	2.870097	-0.342480
6	-3.383739	3.937702	-1.283169
1	-2.843172	3.856491	-2.222256
1	-4.464248	3.872499	-1.451268
1	-3.204784	4.904743	-0.804079
6	-3.578234	2.814018	1.017790
1	-4.285692	3.623173	1.175743
1	-4.064510	1.846195	1.173226
1	-2.767924	2.886217	1.750960
6	6.525426	1.241042	-0.964736
1	6.509180	1.135741	-2.053615
1	6.781756	2.275529	-0.730792
1	7.319830	0.594037	-0.585761

IN21-3
E(RwB97XD) = -1701.61879635 A.U.

6	0.978522	0.347949	-0.061621
6	0.385244	1.167369	1.088402
7	0.630531	-1.055537	0.152836
1	0.475713	0.676907	-0.977924

1	-0.636430	0.857130	1.287490
1	0.977668	1.106530	1.994263
16	-0.383793	-1.721290	-0.935834
8	-0.028808	-1.279089	-2.282055
8	-0.459157	-3.146444	-0.674804
6	-1.985312	-0.998150	-0.594688
6	-2.474197	-1.018971	0.709492
6	-3.714002	-0.460044	0.972409
6	-4.483700	0.109999	-0.048226
6	-3.976731	0.103303	-1.346231
6	-2.730422	-0.447168	-1.627545
1	-1.878007	-1.457056	1.502757
1	-4.094447	-0.465402	1.988306
1	-4.557682	0.539617	-2.151336
1	-2.333210	-0.440339	-2.634783
5	1.154692	-1.853130	1.407038
6	2.471227	0.542653	-0.298515
6	3.249956	1.437598	0.426915
6	3.071044	-0.205307	-1.312200
6	4.605548	1.583952	0.146327
1	2.821811	2.031238	1.224919
6	4.421321	-0.060044	-1.593480
1	2.468612	-0.908378	-1.876072
6	5.195661	0.836730	-0.863072
1	5.199436	2.280891	0.726737
1	4.872094	-0.651520	-2.382232
1	6.252220	0.947969	-1.078466
9	1.859508	-0.923430	2.201828
9	0.066868	-2.353031	2.150933
9	2.002138	-2.892063	1.032844
8	0.409551	2.593951	0.733317
6	-0.587534	3.235755	0.305350
6	-0.341561	4.645223	-0.033330
1	0.682953	4.940064	0.177947
1	-0.572810	4.779397	-1.095902
1	-1.055319	5.266048	0.516590
6	-1.925339	2.634540	0.143254
1	-2.617702	3.356025	-0.281820
1	-1.870119	1.752178	-0.502481
1	-2.303328	2.298088	1.114271
6	-5.834048	0.698684	0.256101
1	-6.192607	1.319030	-0.566577
1	-6.566751	-0.096254	0.422204
1	-5.801959	1.307832	1.162261

TSrot-2
E(RwB97XD) = -1701.61886950 A.U.
Imaginary frequency -24.092 cm-1

8	-1.913267	1.920863	-0.594162
6	-1.843241	0.547901	-0.014157
6	-0.559012	-0.076695	-0.583611
7	0.147637	-0.813467	0.457618
1	-1.728574	0.648879	1.058650
1	0.056659	0.711347	-1.027761
1	-0.805726	-0.777588	-1.378356
16	1.169047	0.095183	1.350989

8	0.594170	1.444677	1.409492
8	1.456363	-0.576156	2.604221
6	2.688592	0.232672	0.429708
6	3.523908	-0.877854	0.335633
6	4.689521	-0.779250	-0.403186
6	5.036019	0.408175	-1.057059
6	4.180521	1.501784	-0.948494
6	3.004787	1.421970	-0.209881
1	3.255819	-1.803568	0.829013
1	5.345386	-1.640150	-0.477420
1	4.434111	2.432745	-1.443912
1	2.347756	2.277316	-0.116792
5	0.376750	-2.329606	0.121923
6	-3.124426	-0.151644	-0.354605
6	-3.577592	-0.217988	-1.672619
6	-3.845558	-0.776333	0.657607
6	-4.750350	-0.896350	-1.968482
1	-3.015772	0.261748	-2.466722
6	-5.013741	-1.468140	0.356502
1	-3.486570	-0.735980	1.680122
6	-5.468628	-1.524372	-0.954211
1	-5.102178	-0.942809	-2.992310
1	-5.566418	-1.960816	1.147525
1	-6.380861	-2.060236	-1.189240
9	-0.892968	-2.919883	0.019313
9	1.027884	-2.428141	-1.126699
9	1.144429	-2.954340	1.104553
6	-2.062165	2.964059	0.095312
6	-2.050651	4.230872	-0.655384
1	-1.884168	4.070798	-1.717184
1	-3.002954	4.741907	-0.482287
1	-1.273601	4.873040	-0.228797
6	-2.225580	2.949468	1.561009
1	-2.418757	3.951838	1.933095
1	-3.040418	2.280287	1.849141
1	-1.296412	2.560283	1.996763
6	6.303115	0.485643	-1.864909
1	6.235406	-0.157006	-2.747339
1	6.499545	1.504457	-2.202115
1	7.160415	0.145571	-1.278779

TSrot-3
E(RwB97XD) = -1701.61050830 A.U.
Imaginary frequency -26.6426 cm-1

6	-0.617750	0.085842	0.098807
6	-0.431119	0.966296	-1.176706
7	0.079564	-1.188007	-0.068811
8	-1.713277	1.494629	-1.641942
1	-0.102991	0.623814	0.897022
1	0.248705	1.799020	-1.007476
1	-0.095831	0.373029	-2.015757
16	1.253781	-1.446784	1.040890
8	0.769856	-1.085986	2.369703
8	1.787839	-2.780216	0.838723
6	2.518150	-0.249275	0.628852
6	3.073223	-0.273175	-0.648491

6	4.041944	0.659482	-0.977514
6	4.468423	1.619134	-0.052408
6	3.893323	1.621889	1.216315
6	2.919274	0.691769	1.564667
1	2.734355	-1.006769	-1.370869
1	4.476912	0.646554	-1.971380
1	4.208119	2.359724	1.946422
1	2.472592	0.695472	2.551096
5	-0.130158	-2.183659	-1.269335
6	-2.059767	-0.023878	0.580363
6	-3.039086	-0.740849	-0.111247
6	-2.422613	0.656442	1.741816
6	-4.349200	-0.757198	0.345763
1	-2.772080	-1.271197	-1.014436
6	-3.736964	0.643953	2.199157
1	-1.669586	1.199376	2.304352
6	-4.705674	-0.060000	1.497564
1	-5.098437	-1.316834	-0.202287
1	-3.996640	1.178058	3.105669
1	-5.731151	-0.074607	1.848200
9	-1.041893	-1.569479	-2.168615
9	1.078710	-2.394419	-1.956620
9	-0.661898	-3.395336	-0.834213
6	-2.330455	2.437749	-1.076428
6	-3.720462	2.631739	-1.516008
1	-3.902261	2.167807	-2.482550
1	-4.332772	2.119598	-0.758025
1	-3.996271	3.685792	-1.503398
6	-1.739491	3.325078	-0.056712
1	-2.509519	3.635653	0.648716
1	-0.886475	2.906989	0.468545
1	-1.414894	4.223869	-0.598577
6	5.538989	2.609151	-0.423993
1	6.516566	2.119165	-0.455561
1	5.355078	3.034152	-1.413460
1	5.595834	3.425018	0.298245

IN22-2

E(RwB97XD) = -1701.63282534 A.U.

7	0.195896	-0.080240	0.405356
6	-1.834717	2.368215	-0.153942
6	-2.270240	0.014211	0.317595
6	-1.006981	-0.804897	0.046908
1	-2.288674	0.336998	1.354979
1	-0.985181	-1.104538	-1.001549
1	-1.077582	-1.708782	0.656477
16	1.070578	0.643913	-0.766247
8	0.528110	0.219283	-2.056953
8	1.138772	2.085743	-0.536410
6	2.714024	-0.013489	-0.605434
6	3.794668	0.845126	-0.491944
6	5.074683	0.309843	-0.419960
6	5.281470	-1.068044	-0.455296
6	4.169848	-1.909055	-0.567619
6	2.888053	-1.392161	-0.643576
1	3.632934	1.914406	-0.450531

1	5.924652	0.977445	-0.330316
1	4.312894	-2.983952	-0.592365
1	2.032725	-2.053371	-0.713576
5	0.685703	-0.184094	1.880671
6	-3.541570	-0.684481	-0.060418
6	-3.794617	-1.038355	-1.386212
6	-4.460125	-1.017440	0.930972
6	-4.960572	-1.715968	-1.712957
1	-3.086895	-0.773685	-2.164137
6	-5.621725	-1.707382	0.603091
1	-4.266311	-0.741569	1.961970
6	-5.873426	-2.054593	-0.718333
1	-5.156382	-1.983705	-2.744540
1	-6.330974	-1.967679	1.379812
1	-6.781258	-2.587828	-0.975501
9	-0.409309	0.163644	2.723235
9	1.053928	-1.505297	2.165583
9	1.745936	0.693427	2.097719
8	-2.169355	1.216943	-0.548462
6	-1.729205	3.388595	-1.209653
1	-2.074566	3.012665	-2.169100
1	-2.276574	4.283945	-0.905267
1	-0.668422	3.656154	-1.267292
6	-1.605225	2.719277	1.259728
1	-1.137110	3.697868	1.326964
1	-2.588811	2.767636	1.745336
1	-1.011442	1.969059	1.782154
6	6.665212	-1.649127	-0.346830
1	6.805434	-2.465426	-1.058960
1	7.430153	-0.893408	-0.531746
1	6.829819	-2.056053	0.655314

IN22-3
E(RwB97XD) = -1701.62008109 A.U.

7	-0.877349	-0.273464	0.050769
6	-3.530609	-1.187312	-0.154621
6	-1.487233	0.956818	-0.460758
6	-2.930619	1.132320	0.082537
1	-1.565316	0.827885	-1.537301
1	-2.962517	1.733480	0.983413
1	-3.585467	1.559957	-0.676428
16	-0.070031	-1.213374	-1.044174
8	-0.516789	-2.594398	-0.910711
8	-0.216499	-0.597304	-2.362746
6	1.647576	-1.151052	-0.597654
6	2.329588	0.053321	-0.735407
6	3.665539	0.109223	-0.384787
6	4.331138	-1.019829	0.106252
6	3.619421	-2.209597	0.238149
6	2.275056	-2.283843	-0.108701
1	1.819461	0.939200	-1.095273
1	4.203469	1.045923	-0.484570
1	4.117622	-3.092856	0.622514
1	1.718878	-3.205590	0.002513
5	-0.569101	-0.479173	1.586996
6	-0.681784	2.231375	-0.264030

6	-0.457035	2.798918	0.990342
6	-0.123887	2.836933	-1.389596
6	0.318458	3.944096	1.111428
1	-0.870746	2.333999	1.875890
6	0.653673	3.983398	-1.268806
1	-0.287757	2.397574	-2.368273
6	0.877054	4.538935	-0.015361
1	0.491337	4.371858	2.092355
1	1.083218	4.439508	-2.153354
1	1.483517	5.431882	0.084751
9	0.689894	0.020396	1.932052
9	-1.553766	0.216353	2.315771
9	-0.634108	-1.849065	1.878179
8	-3.492172	-0.132300	0.538165
6	-3.674375	-2.431338	0.615286
1	-4.115703	-2.243629	1.591749
1	-4.205305	-3.198768	0.055425
1	-2.635623	-2.763983	0.765791
6	-3.492867	-1.228557	-1.626601
1	-2.800520	-2.011305	-1.942920
1	-4.495101	-1.549172	-1.935652
1	-3.254918	-0.285128	-2.106346
6	5.787070	-0.936743	0.476649
1	6.132027	-1.858775	0.946627
1	6.400260	-0.757381	-0.411097
1	5.965171	-0.110121	1.168987

TS22-2
E(RwB97XD) = -1701.62517964 A.U.
Imaginary frequency -180.5239 cm-1

6	2.259768	-0.209154	0.458872
6	0.791789	0.245861	0.370901
1	2.439669	-0.725734	1.400549
1	0.708304	0.937331	-0.465541
1	0.506536	0.751430	1.292521
7	-0.033278	-0.950793	0.144517
16	-1.144993	-0.750808	-1.154695
8	-0.403087	-0.101133	-2.224249
8	-1.739923	-2.051814	-1.374745
6	-2.408860	0.375300	-0.628887
6	-3.602845	-0.131196	-0.131820
6	-4.595485	0.754739	0.250742
6	-4.406643	2.135862	0.156704
6	-3.198402	2.613870	-0.352692
6	-2.196147	1.742573	-0.752049
1	-3.748914	-1.200251	-0.049719
1	-5.533700	0.367618	0.632282
1	-3.041495	3.682360	-0.449163
1	-1.275162	2.124298	-1.173951
5	-0.672158	-1.471459	1.549682
6	3.240355	0.909668	0.270867
6	3.366372	1.540441	-0.967045
6	4.001025	1.349630	1.349376
6	4.251306	2.598017	-1.121123
1	2.778472	1.195377	-1.810515
6	4.878876	2.417437	1.196673

1	3.908177	0.857733	2.311847
6	5.006294	3.040387	-0.038392
1	4.351120	3.080365	-2.086451
1	5.466732	2.756640	2.041473
1	5.694383	3.868989	-0.159878
9	0.385614	-1.477101	2.469804
9	-1.632237	-0.557402	1.972721
9	-1.210162	-2.734054	1.377073
8	2.363098	-1.125758	-0.643367
6	1.572823	-2.171470	-0.590532
6	1.252938	-2.729970	-1.934797
1	1.120644	-1.942037	-2.670963
1	2.109806	-3.349491	-2.220441
1	0.371619	-3.366346	-1.887506
6	1.681030	-3.161759	0.521430
1	0.768289	-3.748688	0.588311
1	2.501445	-3.825908	0.228109
1	1.898118	-2.727924	1.489761
6	-5.477998	3.086647	0.614549
1	-5.411100	4.042506	0.092412
1	-6.473151	2.668906	0.451992
1	-5.372979	3.283946	1.685783

TS22-3
E(RwB97XD) = -1701.61674386 A.U.
Imaginary frequency -189.6058 cm-1

6	-1.435212	1.050748	-0.533711
6	-2.870841	1.438325	-0.071268
7	-1.125761	-0.301276	-0.008487
1	-1.467505	0.942160	-1.614560
1	-2.868731	2.149463	0.748335
1	-3.439159	1.844637	-0.908711
16	-0.236345	-1.292006	-1.090388
8	-0.726607	-2.645666	-0.904225
8	-0.343585	-0.688877	-2.412645
6	1.467255	-1.245502	-0.606097
6	2.269916	-0.208107	-1.061176
6	3.597223	-0.168497	-0.668230
6	4.130939	-1.153034	0.166181
6	3.299267	-2.186828	0.599520
6	1.966477	-2.240204	0.222926
1	1.867095	0.556872	-1.710858
1	4.229066	0.642037	-1.014384
1	3.697824	-2.958167	1.249186
1	1.317398	-3.029584	0.576411
5	-0.680125	-0.439716	1.543299
6	-0.448177	2.174720	-0.269610
6	-0.280792	2.766938	0.982088
6	0.287805	2.661507	-1.348386
6	0.622649	3.806306	1.150148
1	-0.840574	2.401253	1.833334
6	1.194215	3.702810	-1.182195
1	0.152642	2.217987	-2.329927
6	1.365646	4.275262	0.071026
1	0.749369	4.251560	2.130235
1	1.760591	4.065592	-2.032149

1	2.070664	5.087278	0.207740
9	0.582857	0.107651	1.738182
9	-1.624701	0.255153	2.303945
9	-0.676192	-1.795212	1.863928
8	-3.524173	0.284710	0.470030
6	-3.275168	-0.858836	-0.085035
6	-3.466587	-2.033042	0.797154
1	-3.345895	-1.753089	1.839555
1	-4.476856	-2.418450	0.622989
1	-2.751835	-2.807097	0.520318
6	-3.445154	-1.046842	-1.554178
1	-2.975350	-1.970291	-1.882850
1	-4.528828	-1.146570	-1.690942
1	-3.106752	-0.212511	-2.161736
6	5.580549	-1.113970	0.565193
1	5.743606	-1.624233	1.515918
1	6.195132	-1.612969	-0.190413
1	5.937755	-0.086472	0.655761

IN23-2
E(RwB97XD) = -1701.63894217 A.U.

6	0.704803	1.989805	0.925025
6	0.679053	0.475540	1.251713
1	1.080308	2.531053	1.800236
1	-0.318221	0.046887	1.232644
1	1.111226	0.266252	2.225310
7	1.556440	-0.166065	0.229396
16	0.541005	-0.707183	-1.254002
8	0.187676	0.500249	-1.961820
8	1.341331	-1.725986	-1.884305
6	-0.962170	-1.389631	-0.610610
6	-1.063909	-2.758317	-0.382315
6	-2.286580	-3.272496	0.010823
6	-3.402405	-2.446334	0.178132
6	-3.272180	-1.081367	-0.081419
6	-2.061649	-0.543756	-0.487815
1	-0.204702	-3.400230	-0.508623
1	-2.378715	-4.338050	0.188556
1	-4.129545	-0.426802	0.024542
1	-1.976006	0.513161	-0.708968
5	2.352998	-1.515987	0.880669
6	-0.649431	2.546602	0.551693
6	-0.908247	3.069661	-0.709444
6	-1.672581	2.499699	1.499008
6	-2.186690	3.516473	-1.028950
1	-0.114837	3.096494	-1.443261
6	-2.949188	2.939973	1.177495
1	-1.476361	2.105284	2.491751
6	-3.210826	3.445838	-0.092386
1	-2.382564	3.914265	-2.018171
1	-3.739400	2.888617	1.917612
1	-4.207404	3.787539	-0.347301
9	2.727351	-1.175911	2.168276
9	1.416473	-2.530341	0.901693
9	3.436247	-1.781615	0.076930
8	1.619217	2.114104	-0.147927

6	2.498286	1.023370	-0.150791
6	3.158099	0.896497	-1.511695
1	2.443149	0.994871	-2.323278
1	3.888865	1.701184	-1.598923
1	3.679653	-0.056442	-1.581953
6	3.567788	1.199333	0.928786
1	4.307564	0.403146	0.883513
1	4.055795	2.150810	0.712796
1	3.160012	1.242271	1.936612
6	-4.712527	-3.029779	0.626458
1	-4.628728	-3.409781	1.648368
1	-5.510383	-2.286931	0.602685
1	-5.000156	-3.869065	-0.011150

IN23-3
E(RwB97XD) = -1701.62886167 A.U.

6	-1.458699	1.189158	-0.443320
6	-2.777778	1.633026	0.191549
7	-1.360802	-0.268469	0.008572
1	-1.596943	1.128990	-1.519315
1	-2.626527	2.140728	1.143088
1	-3.311713	2.296162	-0.493605
16	-0.350195	-1.256826	-1.180676
8	-0.868673	-2.600096	-1.073825
8	-0.426816	-0.528177	-2.430057
6	1.319216	-1.240766	-0.617663
6	2.173942	-0.241603	-1.064645
6	3.494198	-0.265495	-0.651203
6	3.968357	-1.276612	0.187656
6	3.086093	-2.277933	0.599612
6	1.759987	-2.271148	0.204289
1	1.819248	0.537667	-1.723203
1	4.167891	0.514473	-0.987517
1	3.441100	-3.072729	1.245563
1	1.075090	-3.036930	0.539317
5	-0.759899	-0.480742	1.594804
6	-0.313912	2.148864	-0.220751
6	-0.006137	2.695851	1.025430
6	0.379125	2.600760	-1.342778
6	1.000597	3.641589	1.146161
1	-0.538118	2.370841	1.909833
6	1.387888	3.549881	-1.224736
1	0.128939	2.200884	-2.320152
6	1.704547	4.067595	0.023714
1	1.236036	4.050343	2.121815
1	1.919023	3.884754	-2.108024
1	2.489731	4.808173	0.123540
9	0.500442	0.066512	1.644335
9	-1.635095	0.179581	2.431178
9	-0.723013	-1.843073	1.812341
8	-3.522405	0.456562	0.445229
6	-2.905166	-0.671144	-0.099669
6	-3.292655	-1.867401	0.745821
1	-3.117575	-1.673495	1.799716
1	-4.361963	-2.024910	0.592470
1	-2.754367	-2.759204	0.434607

6	-3.362612	-0.865646	-1.548137
1	-3.010947	-1.806680	-1.966379
1	-4.452059	-0.894163	-1.506680
1	-3.078009	-0.049392	-2.209851
6	5.410261	-1.304012	0.610258
1	5.536500	-1.835730	1.554510
1	6.013414	-1.816994	-0.145118
1	5.808118	-0.293857	0.721195

9

E(RwB97XD) = -1377.05902312 A.U.

6	1.984181	0.283881	-1.122589
6	0.446206	0.093656	-1.028637
1	2.274861	0.399811	-2.172490
1	0.201824	-0.776912	-0.421600
1	-0.002029	-0.038341	-2.014142
7	-0.047837	1.337139	-0.409835
16	-0.934002	1.137465	0.992655
8	-0.181426	0.459894	2.043355
8	-1.534613	2.423558	1.306017
6	-2.195392	0.026885	0.415170
6	-3.051533	0.444559	-0.599444
6	-4.049699	-0.413128	-1.026994
6	-4.210047	-1.678726	-0.452070
6	-3.338208	-2.067241	0.563579
6	-2.327516	-1.221483	1.003503
1	-2.930480	1.421382	-1.051699
1	-4.718159	-0.098104	-1.820815
1	-3.445340	-3.046065	1.017515
1	-1.646739	-1.525838	1.788647
6	2.776035	-0.842355	-0.509795
6	2.728024	-1.057456	0.868428
6	3.539755	-1.686978	-1.307892
6	3.435891	-2.107786	1.434651
1	2.127070	-0.401437	1.489010
6	4.246050	-2.744157	-0.740551
1	3.586718	-1.518386	-2.379137
6	4.196417	-2.954595	0.631211
1	3.395304	-2.268865	2.506071
1	4.838934	-3.397139	-1.370898
1	4.748912	-3.774500	1.076039
8	2.238199	1.484220	-0.412931
6	1.086294	2.307950	-0.472805
6	1.201510	3.298487	0.670980
1	1.286500	2.782837	1.627893
1	2.113986	3.874011	0.508301
1	0.350797	3.976022	0.697866
6	0.990148	3.024418	-1.818708
1	0.104745	3.661871	-1.833547
1	1.879131	3.640366	-1.967424
1	0.914345	2.316283	-2.646564
6	-5.316438	-2.586494	-0.914565
1	-5.392527	-2.588299	-2.004082
1	-5.158792	-3.611320	-0.575801
1	-6.277519	-2.244794	-0.519132

30
E(RwB97XD) = -1377.06804690 A.U.

6	1.387240	1.406535	0.553999
6	2.456965	2.112250	-0.296631
7	1.681528	0.010139	0.206814
1	1.578658	1.576389	1.615146
1	2.114648	3.065610	-0.693976
1	3.371656	2.266867	0.285666
16	1.068623	-1.229182	1.084918
8	1.792552	-2.426985	0.692444
8	1.041181	-0.824881	2.481332
6	-0.620458	-1.394271	0.553643
6	-1.630752	-0.794938	1.293386
6	-2.927790	-0.819083	0.806313
6	-3.228849	-1.429058	-0.411482
6	-2.198630	-2.048952	-1.120899
6	-0.895164	-2.032904	-0.648785
1	-1.400369	-0.305582	2.230308
1	-3.717117	-0.341748	1.376379
1	-2.417622	-2.547342	-2.058943
1	-0.103712	-2.516006	-1.207905
6	-0.030718	1.830845	0.229826
6	-0.598579	1.552688	-1.013327
6	-0.786090	2.504928	1.183510
6	-1.904615	1.927659	-1.288702
1	-0.016529	1.024355	-1.759066
6	-2.094709	2.889267	0.907321
1	-0.355367	2.716003	2.157324
6	-2.658166	2.595851	-0.327187
1	-2.340463	1.692374	-2.253119
1	-2.675532	3.406859	1.662351
1	-3.681658	2.882320	-0.541088
8	2.685131	1.237449	-1.385601
6	2.688907	-0.089289	-0.895568
6	2.256202	-0.990550	-2.037172
1	1.250416	-0.730046	-2.368783
1	2.948029	-0.853894	-2.870111
1	2.279513	-2.035872	-1.730783
6	4.065332	-0.468640	-0.356392
1	4.042403	-1.481629	0.045586
1	4.795146	-0.421451	-1.167358
1	4.373593	0.212362	0.438847
6	-4.628826	-1.386655	-0.959041
1	-4.795550	-2.175884	-1.693935
1	-5.368838	-1.490454	-0.162912
1	-4.808692	-0.426048	-1.452307

M06-2X/6-311G(d,p)computed total energies in DCM, single imaginary frequency, and Cartesian coordinates of the reagents and TSs involved in the LA-catalyzed aziridine ring-opening of 2PMA **19** in presence of ketone **8**, in the absence and in presence of the BH_3 and BF_3 LAs.

2PMA **19**
E(RM062X) = -952.776986542 A.U.

6	-0.117631	-0.694631	0.378080
6	0.589649	-0.379061	1.650904
1	0.191716	-1.628787	-0.084369
1	1.321541	-1.103111	1.992371
1	0.080007	0.214909	2.399805
7	0.899269	0.360683	0.425934
16	2.349986	-0.034046	-0.354735
8	2.822877	-1.332040	0.097523
8	2.149437	0.209277	-1.764048
6	3.408368	1.222302	0.325099
6	-1.522852	-0.274654	0.121900
6	-2.479186	-1.245107	-0.169735
6	-1.904122	1.063330	0.203462
6	-3.806579	-0.884261	-0.367842
1	-2.182150	-2.285794	-0.244641
6	-3.231259	1.422199	0.003900
1	-1.149914	1.815838	0.401926
6	-4.185719	0.450473	-0.279326
1	-4.543019	-1.644972	-0.597069
1	-3.520251	2.464800	0.061612
1	-5.219279	0.733368	-0.437953
1	3.444920	1.097470	1.405556
1	4.392361	1.062188	-0.112948
1	3.006871	2.192594	0.043489

2PMA:BH_3 **9:**BH_3
E(RM062X) = -979.413699381 A.U.

6	0.168179	0.689015	-0.621236
6	-0.533121	1.815693	0.038333
1	-0.197689	0.462666	-1.620416
1	-1.283583	2.340446	-0.539317
1	-0.046827	2.343675	0.848672
7	-0.868372	0.433210	0.421080
16	-2.306234	-0.278276	-0.296914
8	-2.773922	0.666888	-1.290131
8	-1.924555	-1.613204	-0.681315
6	-3.450364	-0.324650	1.057414
5	-0.505128	-0.061909	1.948792
6	1.565372	0.260433	-0.343280
6	2.573816	1.195556	-0.143973
6	1.857277	-1.102266	-0.320464
6	3.878582	0.770306	0.083193
1	2.340520	2.254434	-0.163703
6	3.157912	-1.523638	-0.084502
1	1.055983	-1.820025	-0.463501
6	4.169588	-0.587899	0.115806

1	4.664213	1.499327	0.239659
1	3.383425	-2.582440	-0.052262
1	5.184328	-0.919482	0.299961
1	-4.377884	-0.685523	0.612730
1	-3.065812	-1.005452	1.811295
1	-3.566667	0.683425	1.448385
1	-0.567090	-1.265704	1.932700
1	0.595535	0.369155	2.158200
1	-1.350089	0.470222	2.630530

2PMA:BF_3 **9:**BF_3
E(RM062X) = -1277.36133456 A.U.

6	0.307865	-0.981438	-0.755838
6	-0.416301	-0.320655	-1.864614
1	-0.010336	-2.004579	-0.565801
1	-1.137208	-0.909571	-2.417356
1	0.036013	0.532841	-2.354398
7	-0.789971	-0.014113	-0.469415
16	-2.184717	-0.847336	0.228064
8	-2.669908	-1.729384	-0.810630
8	-1.709601	-1.375709	1.480515
6	-3.354456	0.452484	0.515979
5	-0.515917	1.589296	0.018108
6	1.684791	-0.657090	-0.289670
6	2.712296	-0.443559	-1.199924
6	1.933734	-0.610156	1.080002
6	3.997949	-0.181840	-0.740424
1	2.507433	-0.474764	-2.264289
6	3.216865	-0.339912	1.534613
1	1.116407	-0.765626	1.776718
6	4.249370	-0.127991	0.625426
1	4.799756	-0.012125	-1.448494
1	3.410999	-0.289651	2.598861
1	5.249698	0.083996	0.983049
1	-2.931825	1.129787	1.253804
1	-4.233605	-0.066644	0.897901
1	-3.559051	0.957007	-0.423865
9	-0.600495	1.553775	1.382147
9	-1.557092	2.264831	-0.580127
9	0.697582	1.918138	-0.500038

ketone **8**
E(RM062X) = -193.115148828 A.U.

8	-0.000009	1.390896	-0.000159
6	0.000001	0.187666	-0.000038
6	1.285941	-0.612364	0.002280
1	1.382468	-1.149164	-0.945438
1	2.133438	0.057777	0.129171
1	1.272426	-1.360964	0.797829
6	-1.285933	-0.612378	-0.002143
1	-1.272310	-1.361433	-0.797256
1	-1.382595	-1.148625	0.945877
1	-2.133415	0.057693	-0.129508

TS3
E(RM062X) = -1145.82699091 A.U.
Imaginary frequency -253.6966 cm-1

6	0.103249	0.408981	-0.313410
6	-0.812060	0.754759	0.828001
7	-1.703451	-0.378694	0.791272
1	-0.417392	0.517360	-1.260888
1	-1.259957	1.740254	0.642833
1	-0.286048	0.781870	1.788984
16	-2.953167	-0.183295	-0.191345
8	-2.639030	0.740761	-1.300941
8	-3.518285	-1.481432	-0.523508
6	-4.209636	0.675808	0.767394
6	1.032060	-0.752976	-0.213642
6	1.007175	-1.619839	0.880610
6	1.973495	-0.948082	-1.226358
6	1.947118	-2.638488	0.974283
1	0.213895	-1.522488	1.612110
6	2.918966	-1.961194	-1.123283
1	1.963515	-0.300548	-2.097349
6	2.912359	-2.802825	-0.015208
1	1.915934	-3.317587	1.817607
1	3.646638	-2.104443	-1.912875
1	3.641180	-3.600466	0.064202
8	1.123820	1.772366	-0.579760
6	2.088772	2.128446	0.100718
6	2.651056	1.348775	1.243576
1	1.871975	0.870000	1.831679
1	3.270517	0.546419	0.823770
1	3.273356	1.988426	1.866969
6	2.752097	3.412466	-0.274562
1	3.827388	3.246966	-0.378065
1	2.328725	3.812272	-1.192186
1	2.615223	4.124168	0.545061
1	-4.474116	0.045137	1.613259
1	-3.806703	1.630691	1.103634
1	-5.069244	0.836988	0.118830

TS4
E(RM062X) = -1172.48821973 A.U.
Imaginary frequency -244.8336 cm-1

6	-0.221737	0.430588	0.326526
6	0.699649	0.803813	-0.790378
7	1.699321	-0.232079	-0.636123
1	0.195480	0.680361	1.296732
1	1.073954	1.818851	-0.638272
1	0.254764	0.708628	-1.780885
16	2.915566	0.094403	0.419546
8	2.456074	1.183145	1.286975
8	3.390116	-1.133315	1.015957
6	4.237624	0.762844	-0.580742
5	1.868599	-1.396911	-1.728658
6	-1.106475	-0.737842	0.285385

6	-1.249265	-1.527651	-0.859420
6	-1.844696	-1.041339	1.436648
6	-2.139044	-2.594346	-0.851234
1	-0.642851	-1.338002	-1.736735
6	-2.748055	-2.092156	1.431880
1	-1.702781	-0.443743	2.331094
6	-2.894949	-2.868959	0.283599
1	-2.229269	-3.220177	-1.730187
1	-3.319349	-2.322094	2.322603
1	-3.587095	-3.702691	0.282637
8	-1.362808	1.890177	0.472191
6	-2.367412	2.141669	-0.185990
6	-2.849088	1.298677	-1.325192
1	-3.366310	0.427523	-0.906961
1	-3.546637	1.857426	-1.946800
1	-2.019885	0.923633	-1.922166
6	-3.168750	3.350221	0.183160
1	-3.122339	4.064461	-0.643937
1	-4.217433	3.066278	0.302619
1	-2.783107	3.803947	1.092357
1	3.052467	-1.573853	-1.910332
1	1.341214	-2.418955	-1.338846
1	1.320940	-0.970904	-2.736758
1	5.064749	0.976968	0.094099
1	4.511098	0.012208	-1.318070
1	3.881221	1.675627	-1.055217

TS5
E(RM062X) = -1470.44719784 A.U.
Imaginary frequency -247.6665 cm-1

6	-0.583841	-0.629542	-0.409568
6	0.350776	-0.950993	0.703717
7	1.481425	-0.159502	0.248110
1	-0.337658	-1.151623	-1.328398
1	0.549722	-2.022437	0.734310
1	0.035013	-0.586620	1.678638
16	2.534939	-0.890308	-0.798662
8	1.826187	-2.040122	-1.360275
8	3.095524	0.105878	-1.678779
6	3.844636	-1.536056	0.226686
5	1.872579	1.208712	0.992373
6	-1.331703	0.609334	-0.538303
6	-1.343083	1.590999	0.461097
6	-2.080074	0.795546	-1.709442
6	-2.108958	2.733240	0.287617
1	-0.726631	1.486973	1.345917
6	-2.862857	1.927391	-1.865672
1	-2.040629	0.041900	-2.488607
6	-2.874582	2.896325	-0.864072
1	-2.094055	3.506548	1.044823
1	-3.442780	2.068580	-2.768947
1	-3.469380	3.793185	-0.991643
8	-1.988468	-2.008032	-0.164329
6	-2.961498	-1.989848	0.575313
6	-3.212063	-0.899612	1.575112
1	-3.654228	-0.048067	1.046006

1	-3.907667	-1.230307	2.344834
1	-2.282293	-0.555975	2.025056
6	-3.970345	-3.094120	0.475965
1	-3.958301	-3.664112	1.409311
1	-4.970886	-2.666255	0.374272
1	-3.741065	-3.746355	-0.362793
1	4.527584	-2.060088	-0.440358
1	4.323787	-0.692905	0.716774
1	3.406898	-2.224859	0.947200
9	1.418756	2.287312	0.263886
9	1.205423	1.152752	2.233434
9	3.245407	1.214662	1.159340
